# Modifying PCDH19 levels affects cortical interneuron migration

**DOI:** 10.3389/fnins.2022.887478

**Published:** 2022-10-25

**Authors:** Anna Pancho, Manuela D. Mitsogiannis, Tania Aerts, Marco Dalla Vecchia, Lena K. Ebert, Lieve Geenen, Lut Noterdaeme, Ria Vanlaer, Anne Stulens, Paco Hulpiau, Katrien Staes, Frans Van Roy, Peter Dedecker, Bernhard Schermer, Eve Seuntjens

**Affiliations:** ^1^Developmental Neurobiology Group, Animal Physiology and Neurobiology Division, Department of Biology, KU Leuven, Leuven, Belgium; ^2^Laboratory for NanoBiology, Department of Chemistry, KU Leuven, Leuven, Belgium; ^3^Molecular Signaling and Cell Death Unit, Department of Biomedical Molecular Biology, Ghent University, Ghent, Belgium; ^4^VIB Center for Inflammation Research, Ghent, Belgium; ^5^Department II of Internal Medicine and Center for Molecular Medicine Cologne, Faculty of Medicine and University Hospital Cologne, University of Cologne, Cologne, Germany; ^6^Cologne Cluster of Excellence on Cellular Stress Responses in Ageing-Associated Diseases (CECAD), University of Cologne, Cologne, Germany; ^7^Laboratory of Neuroplasticity and Neuroproteomics, Animal Physiology and Neurobiology Division, Department of Biology, Katholieke Universiteit Leuven, Leuven, Belgium; ^8^Department of Biomedical Molecular Biology, Ghent University, Inflammation Research Center, VIB, Cancer Research Institute Ghent (CRIG), Ghent, Belgium; ^9^BioInformatics Knowledge Center (BiKC), Howest University of Applied Sciences, Bruges, Belgium

**Keywords:** interneuron, medial ganglionic eminence, PCDH19-CE, neuronal migration, brain development, neurodevelopmental disorder

## Abstract

PCDH19 is a transmembrane protein and member of the protocadherin family. It is encoded by the X-chromosome and more than 200 mutations have been linked to the neurodevelopmental PCDH-clustering epilepsy (PCDH19-CE) syndrome. A disturbed cell-cell contact that arises when random X-inactivation creates mosaic absence of PCDH19 has been proposed to cause the syndrome. Several studies have shown roles for PCDH19 in neuronal proliferation, migration, and synapse function, yet most of them have focused on cortical and hippocampal neurons. As epilepsy can also be caused by impaired interneuron migration, we studied the role of PCDH19 in cortical interneurons during embryogenesis. We show that cortical interneuron migration is affected by altering PCDH19 dosage by means of overexpression in brain slices and medial ganglionic eminence (MGE) explants. We also detect subtle defects when PCDH19 expression was reduced in MGE explants, suggesting that the dosage of PCDH19 is important for proper interneuron migration. We confirm this finding *in vivo* by showing a mild reduction in interneuron migration in heterozygote, but not in homozygote PCDH19 knockout animals. In addition, we provide evidence that subdomains of PCDH19 have a different impact on cell survival and interneuron migration. Intriguingly, we also observed domain-dependent differences in migration of the non-targeted cell population in explants, demonstrating a non-cell-autonomous effect of PCDH19 dosage changes. Overall, our findings suggest new roles for the extracellular and cytoplasmic domains of PCDH19 and support that cortical interneuron migration is dependent on balanced PCDH19 dosage.

## Introduction

The developing cerebral cortex generates excitatory glutamatergic pyramidal neurons, which project and signal to different regions of the brain. These cells also make local microcircuits with GABAergic interneurons, which provide inhibitory input into these assemblies. The balance between excitation and inhibition needs to be perfectly controlled for the brain to function properly. Unlike the pyramidal neurons that are formed within the cortex and migrate radially to the cortical plate, interneurons originally derive from the medial and caudal ganglionic eminences (MGE and CGE resp.) during embryonic development (Marín, 2013). From there, they migrate tangentially to the cortex, guided by different extrinsic cues that are either repulsive or attractive (Marín et al., 2010). Besides extrinsic cues, neurons also make use of membrane-bound factors to read their environment while migrating (van den Berghe et al., 2014). Defects in the migration of these cells to the cortex lead to a disturbed balance between excitation and inhibition that can ultimately result in severe early-onset epilepsy (van den Berghe et al., 2013).

*PCDH19* is the second most prevalent gene linked to early infantile epileptic encephalopathy (EIEE) and causes EIEE9 (OMIM #300088) (Dibbens et al., 2008; Jamal et al., 2010; Depienne et al., 2011; Duszyc et al., 2015), a disorder first described by Juberg and Hellman (1971). Recently, this disorder has been renamed to PCDH19- Clustering Epilepsy (CE) (Gecz and Thomas, 2020). Patients with PCDH19-CE suffer from epilepsy already early after birth. In addition to suffering from epileptic seizures, 75 percent of these patients also present with variable cognitive impairment (Cappelletti et al., 2015), and psychiatric comorbidities, such as hyperactivity, obsessive-compulsive behavior, and autism, have been most frequently reported (Kolc et al., 2019).

The pattern of inheritance of this disorder is very peculiar. *PCDH19* is coded by the X-chromosome, yet only females have the disorder, whereas carrier fathers do not have seizures. The discovery of several mosaic male patients suffering from the disease (Depienne et al., 2009; Terracciano et al., 2016; Thiffault et al., 2016; Kolc et al., 2019) showed that the co-existence of cells expressing the wild-type PCDH19 and cells without PCDH19 has a detrimental impact on brain function (Depienne et al., 2009; Kolc et al., 2019). This situation occurs naturally in females after random inactivation of the X chromosome, or in males that have a somatic mutation during early development and become mosaic for PCDH19. Depienne et al. postulated that the mosaicism might lead to impaired cell-cell communication or “cellular interference” (Depienne et al., 2012).

Mice heterozygous for PCDH19 showed relatively minor abnormalities in the brain, yet a striking homotypical clustering of the PCDH19 KO and WT cells within the forebrain correlated with impaired network activity (Pederick et al., 2016, 2018). Lamination of the cortex was not affected and included the presence of cortical interneurons in normal numbers (Galindo-Riera et al., 2021). Nevertheless, minor changes have been observed when studying the behavior of these mice, and PCDH19 has been implicated in mossy fiber synapse formation, supporting the idea that PCDH19 has an important role in establishment and plasticity of micro-circuitry (Galindo-Riera et al., 2021; Hoshina et al., 2021).

PCDH19 is a member of the non-clustered δ2-type protocadherins, has 6 cadherin repeats in the extracellular part, and a cytoplasmic tail containing two conserved domains (CM1 and CM2) (Wolverton and Lalande, 2001; Vanhalst et al., 2005; Hulpiau and van Roy, 2009; Kim et al., 2011). It has only limited adhesive capacity through homophilic interactions but becomes strongly adhesive upon interaction with Cdh2 (N-Cadherin) (Biswas et al., 2010; Emond et al., 2011). In zebrafish, PCDH19 knockdown disturbs the convergent movement of cells during the formation of the neural tube, indicating a role in early neural cell migration (Emond et al., 2009). The cytoplasmic domain of PCDH19 interacts with the WAVE regulatory complex through the so-called WIRS motif (Chen et al., 2014). This interaction is of particular importance since the WAVE regulatory complex controls cytoskeletal remodeling (essential during cell migration), and WAVE complex components, such as CYFIP2, are linked to neurodevelopmental disorders (Takenawa and Miki, 2001; Tai et al., 2010; Abekhoukh and Bardoni, 2014; Chen et al., 2014). This cytoplasmic interaction with the WAVE regulatory complex is shared with several other PCDHs, such as the related PCDH17, which plays a role in collective axon extension (Hayashi et al., 2014). The cytoplasmic domain of PCDH19 is proteolytically cleaved off upon neural activation in hippocampal neurons, and relocates to the nucleus to stimulate gene transcription of immediate early genes (Gerosa et al., 2022). Whether such cleavage takes place during embryogenesis has not been shown.

While other studies have assessed PCDH19 migration in hippocampal neurons (Bassani et al., 2018) or in *in vitro* studies in neurospheres derived from cortical neurons (Pederick et al., 2016), to our knowledge, the role of PCDH19 has not been assessed in cortical interneuron migration before.

Here, we tested the hypothesis that PCDH19 is important for cortical interneuron migration. We found that PCDH19 is expressed dynamically during embryogenesis in the regions generating cortical interneurons, as well as in interneurons invading the cortex. By mimicking the PCDH19 dosage imbalance between cells in *ex vivo* assays, we found that a loss of PCDH19 is less detrimental to migration compared to overdosing. In explants, dosage changes of particular PCDH19 subdomains non-autonomously affected migration of non-targeted cells. In addition, we revealed cell-autonomous domain-specific roles of the PCDH19 protein in migration and apoptosis, and confirmed an *in vivo* role for PCDH19 in interneuron migration.

## Materials and methods

### Animals

All animal experiments were conducted in compliance with the European union and Belgian laws in accordance with the guidelines of the Ethical Committee Animal Experimentation of the KU Leuven (P267/2015). Mice were maintained on CD1 Swiss genetic background. The CD1 mice were crossed with transgenic Dlx5/6cre-IRES-eGFP mice strain, which labels telencephalic interneurons green (Dlx5/6-Cre-IRES-eGFP) (Stenman et al., 2003). The mice were kept in a 14/10-h light-dark cycle in a humidity- and temperature-controlled pathogen free animal unit. Timed matings were performed to obtain pregnant female mice, and a plug check was done every day early in the morning to confirm mating. The vaginal plug day was considered as E0.5.Dlx5/6-Cre-IRES-eGFP positive embryos were verified under a fluorescence binocular microscope (SteREO Discovery.V8; Zeiss; Oberkochen; Germany). Dissection of mouse brains was done in cold phosphate-buffered saline (PBS) and fixation in 4% w/v paraformaldehyde (PFA)/PBS for 16–24 h at 4°C. After fixation, samples were washed two times in PBS and then placed into a storage buffer (0.01% w/v thimerosal/PBS) to preserve the brains. In addition, mouse tail biopsy samples (~5 mm) were also collected for DNA extraction and genotyping of the embryos.

### Cre genotyping

PCR reaction was done with a GoTAQ Polymerase (Promega). The used forward and reverse primer sequences are shown in Supplementary Table S1. Initial denaturation was performed at 94°C for 4 min. Cycling was done for 35 cycles with a denaturation at 94°C for 30 s, primer annealing at 58°C for 30 s and elongation at 72°C for 30 s. The final elongation was done for 7 min at 72°C and then cooled down to 4°C. The PCR product was visualized on a 1% agarose gel.

### Cloning of PCDH19 tagged overexpression constructs

PCDH19 FL, ECDTM, ICD, and ICDΔNLS constructs were obtained from a plasmid containing the full-length murine sequence (PCDH19FL) and cloned into a second plasmid containing the green fluorescent protein (eGFP) behind a cytomegalovirus promoter. Primer sequences to generate the PCR products are shown in Supplementary Table S2.

PCDH19ICDeGFP and PCDH19ICDΔNLSeGFP were generated using tail PCR, and PCDH19FLeGFP and PCDH19ECDTMeGFP were generated using dovetail PCR. Finally, all PCDH19eGFP constructs were recloned into a pCAGGS plasmid, expressing IRES-TdTomato.

### Generation of PCDH19-V5 and PCDH19 KO mice

All the primers used to generate the PCDH19-V5 and PCDH19 KO mouse lines were ordered from IDT. These mouse lines were created in the lab of Bernhard Schermer.

#### Single-guide RNA generation

In order to generate the single-guide RNA (sgRNA), a PCR reaction was done. The PCR was done with the Q5 High-Fidelity Polymerase (NEB). The used forward and reverse primer sequences are shown in Supplementary Table S3. As a PCR template, the pSpCas9 (BB)-2APuro (Px459) V 2.0 (Addgene62988) was used. Initial denaturation was performed at 98°C for 30 s. Cycling was done for 30 cycles with a denaturation at 98°C for 10 s, primer annealing at 60°C for 30 s, and elongation at 72°C for 10 s. The final elongation was done for 2 min at 72°C and then cooled down to 10°C.

#### Gel extraction

A 1% gel was done to extract the PCR product. To visualize the PCR reaction, 2 μl loading dye was added to the reaction. The complete PCR product was loaded onto the gel. The electrophoresis was done for 30 min at 90 V. The gel-extracted band had a size of 120 bp. Gel extraction was performed using the Qiagen gel extraction kit according to the manufacturer's instructions. Elution was performed in a 30 μl elution buffer. After elution the concentration was measured using the Nanodrop. In order to verify the extraction, 2 μl of the extracted DNA was loaded into a 1% gel.

#### *In vitro* transcription

The HiScribe T7 High-Yield RNA Synthesis Kit (NEBE2040S) was used to transcribe the DNA into RNA. The reaction was set for short transcripts (<0.3 kb). A reaction of 20 μl was set up where the final concentration of the reaction buffer was 0.75X. About 11 μl was used of the template gel-extracted DNA. About 1.5 μl was used from the T7 RNA polymerase mix. The transcription was performed for 16 h overnight at 37°C. To verify if the transcription was successful, 2 μl of the obtained unpurified RNA was loaded on a 2% agarose gel.

#### RNA purification

For the purification, the RNeasy kit (Qiagen) was used. About 700 μl of QIAzol lysis reagent was added to the sample to homogenize. The homogenate was incubated at room temperature for 5 min. After chloroform extraction, samples were loaded on the RNeasy mini column in a 2-ml collection tube. The samples were eluted in 30–50 μl RNAse free water. Concentration of the eluted RNA was measured on a 1:10 diluted sample and verified on a 2% gel.

#### Generation of mouse lines via electroporation of zygotes

Mouse generation was done according to Tröder et al. (2018). For the PCDH19-V5 mouse line, 4-μM sgRNA, 4-μM Cas9protein, and 10-μM ssODN were used. sgRNA was made as described above and the repair template ordered from IDT (sequences in Supplementary Table S3). For the PCDH19 KO mouse line, 4 μM of SpCas9 WT protein was complexed with a mix of 4 different guide RNAs (2 μM each), targeting a large deletion. Guide RNAs were ordered *via* IDT as crRNA (IDT, Alt-RTM crRNA) (sequences in Supplementary Table S3) and used together with the tracrRNA (IDT, 1072532).

### Genotyping

A small ear biopsy was collected at weaning and used as well for identification of the mouse. Ear biopsy was deposited into a sterile Eppendorf tube and incubated with a 200 μl lysis buffer [1-M Tris-HCl (pH = 8.5), 0.5-M EDTA (pH = 8), 10% sodium dodecyl sulfate (SDS), and 5-M NaCl], containing 1:100 Proteinase K (10 μg/μl in 40% glycerol/nuclease free water) at 56°C overnight.

### Pcdh19-V5 and KO Genotyping

PCR reaction was done with a GoTAQ Polymerase. The used forward and reverse primer sequences are shown in Supplementary Table S4. Initial denaturation was performed at 94°C for 3 min. Cycling was done for 34 cycles with a denaturation at 94°C for 30 s, primer annealing at 60°C for 30 s, and elongation at 72°C for 1 min. The final elongation was done for 10 min at 72°C and then cooled down to 10°C. The PCR product was visualized on a 2% agarose gel. Primers are in Supplementary Table S4.

### *In situ* hybridization

A 560 base pair fragment of the mouse *PCDH19* gene exon1 was cloned into a pJET1.2 vector. *In vitro* transcription was done using 1 μg as a template of the linearized plasmid, using the T7 DIG RNA labeling kit (Roche, Sigma-Aldrich). Purification of the RNA was done using the Micro Bio-Spin™ P30 gel columns (Bio-rad) according to the manufacturer's instructions, and RNA concentration was measured using the SimpliNano spectrometer (Biochrom). All the ISH were done on 6-μm paraffin sections using an automated platform (Ventana Discovery, Roche). About 200 ng of dioxigenin labeled probe was diluted in RiboHybe (Roche) and vortexed prior to use. After deparaffination *via* heat, slides underwent pre-treatment with a citrate buffer (pH = 6.) at 95°C and with proteinase K at 37°C for 4 min. The probe in the aforementioned amount was added per slide, and denaturation was performed at 80°C. Next, hybridization was performed for 6 h at 65°C. Several washes had occurred before the anti-DIG antibody conjugated to alkaline phosphatase was added at the concentration of 1:1,000, followed by an incubation of 8 h with the substrate (BlueMap Detection Kit, Roche). Slides were manually dehydrated in a graded ethanol dilution and mounted with Eukitt (Sigma). Brightfield images were acquired using a LeicaDM6 B microscope connected to a digital CMOS camera (DMC2900, Leica) with the LAS Xsoftware suite (Leica). Further processing was done with Fiji and GIMP or Photoshop.

### Hybridization chain reaction

This protocol is an adaptation of the HCR3.0 protocol described in Choi et al. (2018). We refer to buffer compositions described in this protocol, and used the method described in Elagoz et al. (2022) for probe design. To ensure the probe could distinguish WT from KO, we used the ~1,000 bp PCDH19 sequence that was deleted in the PCDH19 KO mice as input for the probe generator. Off-target probes were identified using BlastN and were excluded from the probe set. DNA oligo pools from the designed probes were ordered from Integrated DNA Technologies (IDT), dissolved in UltraPureTM DNase/RNase-Free Distilled Water (Invitrogen) and stored at −20°C. HCR B2 amplifier labeled with Alexa Fluor^®^ 647 was ordered from Molecular Instruments. Components of all used buffers and solutions as well as the sequences for the DNA probe sets are listed in Supplementary Tables S5–S9.

Before starting the protocol, vibratome sections were placed in a 24-well plate (maximum, three sections per well). First, tissues were permeabilized with 600 μl of a permeabilization buffer (1% DMSO, 1% Triton-X in autoclaved PBS) for 2 h at 37°C in a humidified surrounding in the dark. Next, a permeabilization buffer was replaced with a 600 μl pre-warmed probe hybridization buffer, and tissue was incubated for 1 h at 37°C in a humidified surrounding in the dark. In the meantime, HCR probes were thawed on ice, spun, and a 12 nmol DNA probe was diluted in a 200 μl pre-warmed probe hybridization buffer (probe solution) per well or glass slide. After incubation, the probe hybridization buffer was replaced by 200 μl of probe solution. Tissue was incubated for 18 h at 37°C in a humidified surrounding in the dark.

The next day, excess probes were washed from the tissue in several wash steps (600 μl of each wash solution, see Table 1) at 37°C in a humidified surrounding in the dark.

**Table 1:** Probe wash steps for the HCRv3 protocol.

**Step**	**Solution**	**Timing**
1	25% 5X SSCT/75% probe wash buffer pre-warmed at 37°C	15 min
2	50% 5X SSCT/50% probe wash buffer pre-warmed at 37°C	15 min
3	75% 5X SSCT/25% probe wash buffer pre-warmed at 37°C	15 min
4	100% 5X SSCT pre-warmed at 37°C	15 min
5	100% 5X SSCT at RT	5 min

To amplify the probe, the last wash solution was replaced by a 600 μl amplification buffer, and tissue was incubated for at least 30 min at RT in a humidified surrounding in the dark. Next, 9 pmol of both amplification hairpins (H1 and H2) per well or glass slide was pipetted into pcr-tubes, heated at 95°C for 90 s, immediately put on ice for 5 min, and incubated at RT for 30 min in the dark. Next, the hairpins were individually dissolved in the amplification buffer (100 μl per 9 pmol hairpin) before they were combined and mixed well (hairpin solution). The amplification buffer was replaced with 200 μl hairpin solution. Tissue was incubated for 16 h at RT in a humidified surrounding in the dark.

The following day excess hairpins were removed by washing tissue three times with 600 μl of 5X SSCT for 10 min. Next, sections were stained with DAPI and mounted.

A control condition in a well was included in which probe solution was replaced by a 300 μl probe hybridization buffer, and hairpin solution was replaced by a 300 μl amplification buffer. Thus, these sections were not treated with probes and hairpins to control for autofluorescence of the tissue.

### Protein isolation and western blotting

**Figure 1:**
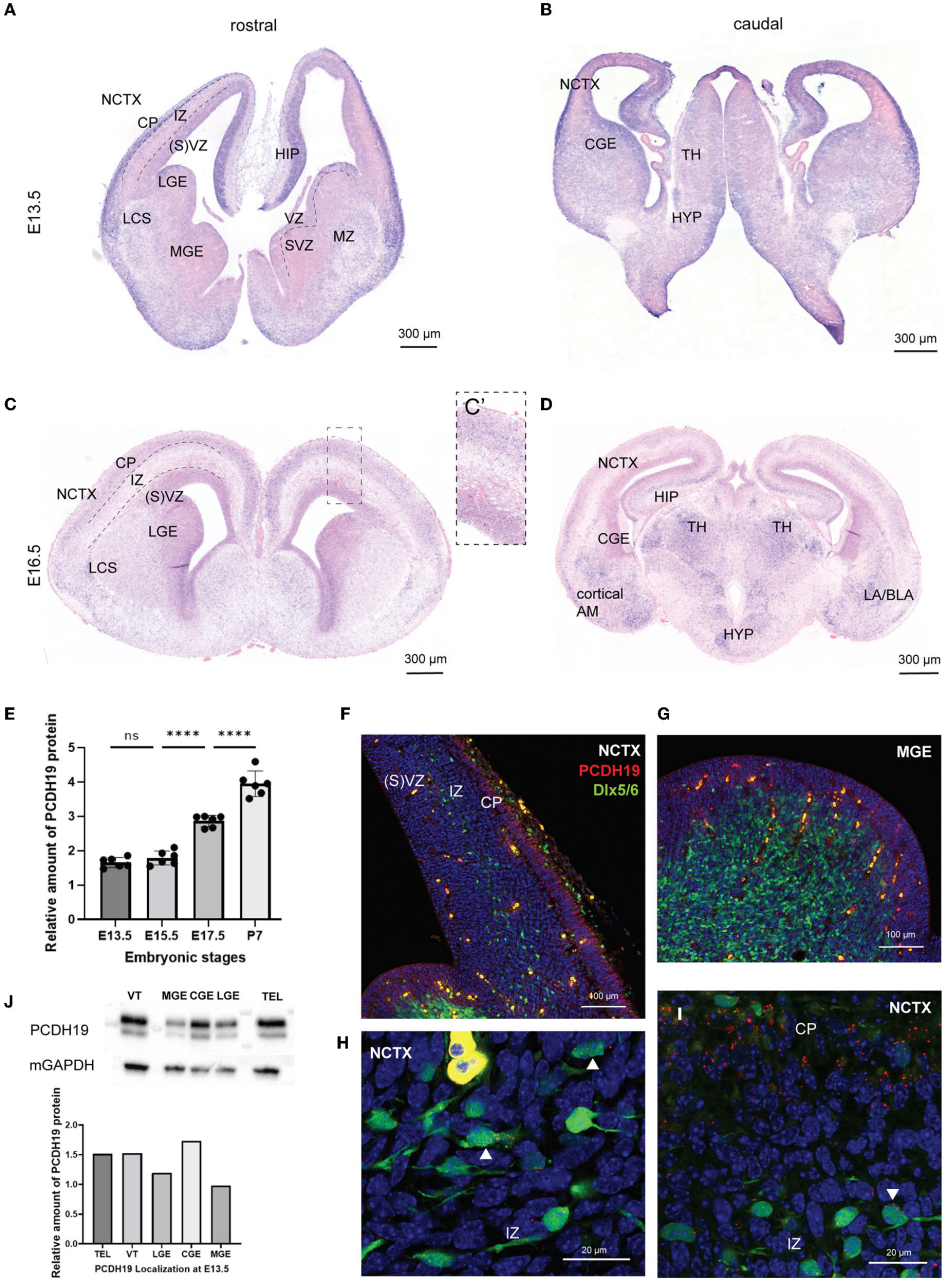
*Pcdh19* is expressed in the developing mouse forebrain and in a subset of the cortical interneurons. **(A–D)** *Pcdh19* mRNA was detected in coronal sections of a wild-type mouse at E13.5 **(A,B)** and E16.5 **(C,D)**. **(A)** At E13.5, ISH of *Pcdh19* showed high expression within the CP, HIP, and, to a lower extent, in the SVZ within the NCTX. In the VT, *Pcdh19* was detected in the GEs within the LGE, MZ, and in the LCS. Sparse expression is detected within the VZ in the VT. **(B)** More caudal expression is also detected within the CGE, TH, and HYP at E13.4. **(C)** At E16.5, mRNA becomes more confined to the CP within the NCTX **(C)**. Scanty expression can still be detected in the SVZ and LCS. **(D)** Caudal expression is found within the limbic system in the TH, the AM (LA, BLA, cortical AM), and the HYP. **(E)** Quantification of PCDH19 protein in lysates of telencephalon collected at different embryonic stages showed a significant increase in the production of PCDH19 at late embryonic and early postnatal stages. **(F–I)** HCR for PCDH19 at E13.5, dots represent RNA molecules. **(F)** PCDH19 was expressed at high levels in the NCTX cortical plate, and at lower levels in the (S)VZ, and is nearly absent from the IZ. **(G)** PCDH19 was expressed at low levels in the (S)VZ and nearly absent from the mantle zone. **(H,I)** Zooms on migrating interneurons showed some expressed PCDH19 stronger (white arrowheads), whereas others were negative. Interneurons migrating in the cortical plate mostly had a low-level expression of PCDH19. **(J)** Western blot and relative quantification of E13.5 mouse VT, TEL, and the distinct GE regions show PCDH19 protein production at the expected molecular weight of nearly 126 kDA in all the lanes. Total protein was assessed with the housekeeping gene mGAPDH. AM, amygdala; BLA, basolateral amygdala; CGE, caudal ganglionic eminence; CP, cortical plate; E, embryonic day; HIP, hippocampus; HYP, hypothalamus; ICD; intracellular domain; IZ, intermediate zone; LA, lateral amygdala; LCS, lateral cortical stream; LGE, lateral ganglionic eminence; MZ, mantle zone; MGE, medial ganglionic eminence; mGAPDH, murine glyceraldehyde 3-phosphate dehydrogenase; NCTX, neocortex; P, postnatal day; SVZ, subventricular zone; TEL, telencephalon; TH, thalamus; VT, ventral telencephalon; VZ, ventricular zone.

Depending on the experiment, MGE, LGE and CGE was dissected out of 10 embryos at stage E13.5 and pooled per brain region (Figure 1J) or individual whole telencephalon (Figures 1E,J), ventral telencephalon (Figure 1J) or entire brain (Supplementary Figure 1F) were isolated. For protein isolation and quantification, the tissue lysates were homogenized in a 100 μl/sample ASBA buffer [1% Triton-X-100, 0.1% SDS, 1-mM EDTA, 50-mM NaCl, 20-mM Trizma base (pH = 7.5)] and 4 μl/sample cocktail protease inhibitor solution (Roche). The homogenization was performed mechanically with a drill for 5-x-10 s/sample and kept on ice. Afterwards, the brain samples were centrifuged for 5 min at 13.000 rpm at 4°C. Supernatants were collected, and the Qubit Protein Assay Kit (Invitrogen) was used to determine protein concentration of the brain lysates.

About 15 μg of the protein lysates was loaded on a precasted 4–12% Criterion XT Bis-Tris 26 well-gel of Biorad and blotted on a PVDF membrane using the Trans Turbo Blot from BioRad (7 min). Next, the membranes were dehydrated in Tris-saline for 5 min and incubated with a blocking buffer for the following 2 h. All the washing and incubation steps occurred at room temperature (RT) and placed on the shaker. After the 2-h incubation, the membrane was incubated with the PCDH19 primary antibody (Bethyl A304-468A) diluted 1/1,000 and left overnight to incubate. The next day, blots were rinsed 3 x 5 min with Tris-saline and incubated with the secondary antibody solution with HRP-labeled antibodies (GAR:Goat Anti-Rabbit) 1/10,000 in the blocking buffer for 30 min. Next, membranes were rinsed with Tris-saline (2 × 5 min) and incubated with Tris-Stock (1 × 5 min). After the Tris-Stock incubation, the Clarity western ECL substrate kit (BioRad) was used to reveal the blots. Imaging of the membranes occurred with the imaging system (BioRad).

The membranes were stripped using a Restore western blot stripping buffer of Thermo Scientific. Next, the membrane was rinsed again with Tris-saline (3 × 5 min) and incubated with the blocking buffer for 2 h. The membranes were incubated overnight with GADPH 1/1,000 (GAPDH Millipore cat nr: MAB374) in the blocking buffer. The next day, a HRP-labeled antibody (Goat Anti-Mouse) 1/25,000 solution was added and, afterwards, imaged with the BioRad imaging system.

### Organotrophic slice electroporation

One day before the actual experiment, Millicell Cell Culture inserts with a pore size of 0.4 μm were coated with poly-L-Lysin (Sigma) and Laminin (Sigma). Coated inserts were placed in PBS into a 6-well plate overnight at 37°C in the cell culture incubator. On the next day, coating solution was removed, and inserts were placed on top of a slice culture medium. Dlx5/6-Cre-IRES-eGFP E13.5 brains were dissected in ice-cold L15++ (a Leibovitz‘s L15 medium, supplemented with glucose and Hepes). Subsequently, the dissected brains were embedded in 4% low-melting point agarose in L15++. Polymerization was obtained after 1 h on ice. Next, coronal slices of 300 μm were obtained with the vibratome and collected in L15++ media. Settings of the vibratome were the following: speed was less than 15, frequency was set at 60, and the amplitude at 0.6. Sectioned slices were placed on top of a membrane and placed on top of an agarose bed, previously prepared into the square well of a Petri dish square platinum electrode (CUY701P20E, Nepagene). Injection was performed under a binocular (Leica) into the MGE. For OE Plasmids, we injected at a concentration of 2 μg/μl. To generate the LOF, clustered regularly interspaced short palindromic repeats (CRISPR-Cas9) components were pre complexed to ribonucleoprotein (RNP) in an Opti-Mem medium for at least 20 min at room temperature injected at the amount of 380 ng for the guide ribonucleic acid (RNA) and 300 ng for the Cas9 protein. All LOF compounds were mixed with pCAGGS plasmid at a concentration of 1 μg/μl in order to trace the electroporated cells. To visualize the injection site, Fast Green was used at an amount of 1 μl per 33 μl. For the negative electrode, an agarose cylinder of 1% was attached to the cover square of platinum plate electrode (CUY701P20L, Nepagene). Electroporation was performed using the following settings: 5 pulses, 150V with a pulse length duration of 5 ms using 100-ms intervals. A BTX electroporator (ECM830, Harvard Apparatus) was used. Subsequently, after electroporation, electroporated sliced were placed on top of the coated inserts and placed on ice. Three slices with the same condition were placed on top of an insert. After all the inserts were covered with slices, the slices were cultivated in the cell culture incubator. After 2 days *in vitro* (div), 500 μl of the used slice culture media was removed and refreshed with new slice culture media. After 3 div slices were fixed in 4% PFA for at least 2 h, stained with DAPI and mounted on microscope slides with Mowiol and imaged with the confocal microscope (Olympus). Analysis was performed using ImageJ software, total amount of TdT positive cells was quantified within every slide side, and the proportion or cells reaching the cortex compared to the total amount was calculated.

Statistical significance was determined using a Kruskal Wallis multicomparison test with Dunn‘s *post hoc* test.

### MGE explants in Matrigel culture

At E13.5 Dlx5/6-Cre-IRES-eGFP embryos were dissected from the mother in L15++ media. Embryos were kept on ice, while the skin and meninges were removed. Subsequently, the neocortex (NCTX) was open up from the top, superficially, in order to expose the GEs. Injection into the MGE was performed using approximately 10 injection sites per MGE. The same injection mixtures and amount of OE plasmids and CRISPR-Cas9 components as used for the slice electroporation (slice EP) were used. Subsequently, after injection in both MGEs, electroporation was performed using the following settings, 5 pulses, 50 V with a pulse length duration of 50 ms using 1-s intervals. Electrodes (CUY650P5 tweezer electrodes) connected to a BTX electroporator (Harvard Apparatus) were placed on each side of the embryonic head at the position of the ears. After electroporation embryos were kept at 4°C for 3 h in L15++. Meanwhile, the matrigel was thawed in ice. Matrigel was diluted in a 1:1 ratio with complete neuro basal media. After the incubation period, MGE was dissected from the embryos, and each MGE was cut into small pieces. Subsequently, these pieces were embedded carefully into the neurobasal media-diluted matrigel and placed as matrigel drops containing the piece into 35-mm glass bottom imaging (Ibidi) dishes. Once placed into the dish, the Matrigel containing explant was allowed to polymerize at 37°C. After polymerization, the explant was surrounded with complete neurobasal media and cultured in the cell culture incubator for 2 days. After 2 div explants were fixed with 4% PFA and imaged with the confocal microscope (Olympus). Stacks were projected with the Image J software, and analysis of migration from the explant was performed using Cell Profiler software (McQuin et al., 2018; Stirling et al., 2021). Statistical significance was determined using the Mann–Whitney U Test for the sample size of 2 and with the Kruskal–Wallis non-parametric test and the Dunn's *post hoc* test for bigger sample sizes.

### Cell profiler analysis migration from the explant

The following cell profiler pipeline for analysis with the following modules was used. The z-projected image of the explant and the red fluorescence channel for the positive TdT cells were used as starting images for the images module. Metadata was extracted from the image file. The explant brightfield image and the TdT cell image were defined with names and types. We used “identify primary objects” as modules to detect the explant and the TdT neurons. The explant was defined with the Cell profiler as one primary object. All the TdT neurons cell bodies were identified as primary objects; the result was verified. Once all objects were identified, we used the two-module mask objects and relate objects. In the relate object module, the explant was set as a parent object and the neuron cell bodies as child objects. Using this module, distance between the parent object and all child objects was measured. Distances were obtained in pixels. Transforming of pixels into μm was achieved using the conversion factor stored in the .oib file using Image J. Finally, Graph Pad Prism 8.3.0 was used to determine the statistical significance.

### MGE explant morphological analysis

Morphological analysis of MGE explant-derived neurons was performed using the SNT ImageJ plugin (Longair et al., 2011; Arshadi et al., 2021). A minimum of 4 explants obtained from at least two experimental replicates were analyzed per treatment group. Means per experimental group were compared with the Kruskal–Wallis non-parametric test and the Dunn's *post hoc* test for a sample size bigger than 2 and with the Mann–Whitney U test for the comparison between two samples. Polarity analysis was compared across experimental conditions using two-way repeated measures ANOVA with Holm-Sidak *post hoc* comparison. Neuronal category frequencies were compared between neuronal morphology and experimental groups using two-way repeated measures ANOVA with Holm-Sidak *post hoc* comparison.

### Cell culture and transfection

N2A cells were grown in DMEM high glucose supplemented with 2-mM L-glutamine, 1% penicillin/streptamycin, and 10% fetal bovine serum albumine (Neuro2A media). For confocal microscopy, we used glass bottom dishes (Ibidi) and pre coated them with geltrex at least 3 h prior seeding; for the rest of applications, we used 6-well plates. One day prior, transfection cells were seeded to a density of 5 × 10^5^ (a 6-well plate) and 5 × 10^4^ (Ibididish) and left to adhere. On the day of the transfection, the cells were checked for adherence and confluency under the microscope. Transfection was performed using Lipofectamine 3000 (Thermo Fisher Scientific). Briefly, Lipofectamine 3000 reagent was diluted in Opti-Mem using the higher amount suggested by the manufacturer for 6-well or 24-well (Ibidi dish) and vortexed and spun down. About 5 μg of plasmid DNA was diluted in Opti-MEM, and, subsequently, the 2 μl of P3000 reagent per 1 μg DNA was added to generate the master mix. The mastermix was spun down and mixed 1:1 with the prior diluted lipofectamine in Opti-Mem. The mixture was spun down and incubated for 30 min at room temperature. After the incubation, mixture was added to one well/dish drop by drop, and the cells were given an easy shake to mix the reagent better into their media. The cells were incubated at 37 °C in an incubator. Transfection efficiency was controlled after 24 and 48 h under a fluorescent microscope.

### GFP Pulldown

About 500 μg of protein per condition was used to start the pull down. The Miltenyi μM ACSGFP isolation kit (130-091-125) was used. About 50 μg GFP beads were incubated with lysate, containing 500 μg protein for 1 h in the cold room with overhead shaking. A μMACS column and magnet were placed in the cold room. About 200 μl of an ice cold lysis buffer containing the proteinase inhibitor cocktail was used to preclear the columns. Whole lysate containing the beads was loaded on the magnetic column. Beads were washed two times with a wash buffer 1 [50 mM Tris (pH = 7.5), 150-mM NaCl, 5% glycerol, and 0.05% Triton-X100]. Then, the beads were washed three times with wash buffer 2 [50-mM Tris (pH = 7.5), 150-mM NaCl]. Elution was conducted *via* adding 20 μl of a 95°C pre-heated elution buffer to the column and incubated at room temperature for 5 min. Subsequently, 50 μl was further eluted through the column, and analysis was done by Western Blot.

For western blot detection after the GFP pull down, proteins were heat denatured in a mixture of sample-reducing agent 10 × (NOVEX, NP0009), and loading dye LDS Sample Buffer 4 × (NOVEX, NP0007) was as well added to the protein. All amounts were calculated for a volume of 20 μl and boiled at 70°C for 10 min. Separation was performed on a precast NuPAGE 4–12% Bis-Tris gel [Invitrogen, (WG1403BX10) with NuPAGE MOPS SDS Running Buffer (NP0001) and immune blotted to a nitrocellulose membrane (Transfer Pack, Midi format, 0.2-μm nitrocellulose, Bio-Rad, 170-4159) using a Trans Blot Turbo system (Bio-Rad)]. Standard protein detection was performed using mouse anti-GFP antibody -HRP (1:5,000; Biorad), rabbit anti-PCDH19 (1:500 Millipore). After 2-h blocking in 5% w/v non-fat dry milk/TBST (a blocking buffer) at RT, o/n incubation at 4°C in a primary antibody diluted in the blocking buffer, and washing in TBST, transfer membranes were incubated during 45 min in HRP-conjugated anti-rabbit secondary antibodies (Bio-Rad) diluted 1:10,000 in a WB buffer. Protein bands were visualized with a ChemiDoc MP imaging system (Bio-Rad) after incubation in ECL substrate (Thermo Fisher Scientific).

### Flow cytometric analysis

To perform our apoptosis analyses, we trypsinated the Neuro2A cells and stopped the enzyme with media-containing serum. In addition, we kept all the washes to not loose dead cells. After centrifugation, the supernatant was discarded, and cell pellets were resuspended in PBS and transferred into micro centrifuge tubes. In order to have a positive control for early and late apoptosis, the untransfected cell condition was used. Half of the cells were killed intentionally for 15 min at 75°C. These cells were then mixed with the rest of untransfected cells in equal parts and spun down. Once the positive apoptosis control was made, all the cell suspensions were spun down and resuspended into a 100 μl Annexin V-binding buffer (BD Biosciences). Annexin V (V450, 560506 BDBiosciences) and 7-aminoactinomycin D (7AAD) (559925 BD Biosciences) were added to analyze for early and late apoptosis, excluding the single-stain controls. The single-stain controls were the positive apoptosis control either incubated with Annexin V or 7AAD. Incubation was performed for 15 min at room temperature in the dark. After the incubation, an Annexin V-binding buffer was added, and cells were passed through tubes with a 35-mm strainer (VWR) and analyzed with the SH800 Cell Sorter (Sony).

### Image acquisition

N2A cells images were acquired using the 60X magnification with oil immersion of the confocal microscope (an Olympus FV1000 microscope) and taking fluorescent z-stacks with a depth of 1–2 μm, a speed of 4 μs/pix and a pixel size of at least 1024 × 1024 pixels. Whole z-depth of the DAPI-channel was covered.

### Quantification of the nuclear-cytoplasmic ratio

For nuclear-cytoplasmic distribution calculations, a single z-slice was manually selected from the provided z-stack in order to have the most central view of the cell through the nucleus and to have the highest number of cells in focus. These images were split in individual channels [DAPI (blue), GFP (green) and TexRed (red)]. Images were segmented and analyzed using Cell Profiler using a custom-made pipeline. Briefly, the cells were segmented by identifying individual nuclei through the DAPI channel using a global Otsu threshold method. Cells edges were identified using a propagation algorithm using the fluorescence channel that would best describe the totality of the cell, leading to a one-to-one correspondence between each nucleus and the respective cell. Nuclear areas were defined by the DAPI stain and the cytoplasmic areas were obtained by subtracting the nuclear areas to the identified total cell areas. Shrinking of 1 pixel of nuclei was used to better define the cytoplasmic areas. After segmentation, the average fluorescence intensity was calculated for each cell in both the nucleus and cytoplasm-identified objects. Image analysis with CellProfiler 2.2.0 and R Studio 3.6.1 was conducted by Marco Dalla Vecchia. Images with cells in a clearly dying state or with many saturated pixels were discarded from the analysis.

### Quantification of the GFP-TdT ratio

Z max projection was made of the explant images. The area of the explant was defined by the Dlx5/6-Cre-IRES-eGFP IN in the GFP channel. The same area was applied to the TdT channel, and the threshold was adjusted before measurement. Intensity measurements were performed with Fiji is just ImageJ and statistical analysis performed with Graph Pad Prism 8.3.

## Results

### *Pcdh19* mRNA and PCDH19 protein are produced in the ganglionic eminences during embryonic mouse brain development

Different studies have shown that *Pcdh19* is expressed in the developing mouse cortex and hippocampus within pyramidal neurons (Kim et al., 2007; Fujitani et al., 2017; Pederick et al., 2018; Gerosa et al., 2022). Only a few groups have investigated *Pcdh19* expression in inhibitory neurons (Bassani et al., 2018; Serratto et al., 2020; Galindo-Riera et al., 2021). We hypothesized that proper migration of inhibitory neurons to the cortex could be affected in PCDH19-CE. To address this question, we first assessed *Pcdh19* expression in developing cortical interneurons neurons by means of *in situ hybridization* (*ISH*). GABAergic cortical interneuron migration occurs from E12-E19 in the developing mouse brain from the ganglionic eminence to the NCTX (Guo and Anton, 2014). We, therefore, studied *Pcdh19* mRNA at E13.5 and E16.5 in the ganglionic eminence and NCTX in mouse embryonic brain slices (Figures 1A–D). At E13.5, *Pcdh19* is clearly expressed in the cortical plate and the ventricular zone within the NCTX, and in the mantle zone (MZ), the dorsal lateral ganglionic eminence (LGE) ventricular zone (VZ), and the lateral cortical stream (LCS) of the ventral telencephalon. Caudally, *Pcdh19* mRNA could be detected throughout the CGE, thalamus (TH), and hypothalamus (HYP) (Figure 1B). At E16.5, *Pcdh19* expression became less prominent in the cortical plate (CP) than at E13.5 (Figure 1C) but appeared more restricted, which could be the prospective layer 5, where *Pcdh19* expression has been described postnatally (Galindo-Riera et al., 2021) (Figure 1C). In the ventral telencephalon, it was still visible within the LCS. Caudally, expression was high in the limbic system within the TH, amygdala (AM) and HYP (Figure 1D). To investigate the temporal dynamics of protein production of PCDH19, we performed western blot (WB) on lysates of whole telencephalon at E13.5, 15.5, 17.5, and P7 and quantified the relative amount of PCDH19 at each stage (Figure 1E, Supplementary Figure 1A for WB). This analysis showed a significant increase in PCDH19 production at late embryonic stages and at P7, in line with the described roles of PCDH19 at the synapse. To further explore the expression of *Pcdh19* in embryonic cortical interneurons, we made use of the Dlx5/6-Cre-IRES-eGFP mouse line that labels migrating cortical interneurons during embryogenesis (Stenman et al., 2003). We focused our analysis on E13.5, given many assays in this manuscript use this time point, and interneuron migration is easy to visualize at this stage. We revealed *Pcdh19* expression by hybridization chain reaction, which enables co-localization and semi-quantitative evaluation of expression *in situ*. The HCR signal appears dotty, and each dot represents at least one mRNA molecule. The number of dots can be used as a proxy for the expression level. This analysis confirmed the colorimetric ISH data shown in Figures 1A,B, and indicated that some, but not all interneurons migrating through the cortex, expressed *Pcdh19* (Figures 1F,H, Supplementary Figure 1F). *Pcdh19* was also expressed in the ventricular zone of the MGE, while it was nearly absent from the mantle zone (Figure 1G). The marginal zone stream of interneurons tends to express *Pcdh19* at a low level in most cells (Figure 1I, Supplementary Figure 1G). We complemented this analysis with a study at the protein level, performing WB on lysates from the whole ventral telencephalon, telencephalon and distinct parts of the ganglionic eminence at E13.5. A strong band around 126 kDa, which is the predicted MW of PCDH19, indicated that PCDH19 was produced in the MGE, CGE, and LGE (Figure 1J). Taken together, these results show that PCDH19 was present in the main regions that generate cortical inhibitory neurons at the assessed time points, and in variable levels in individual interneurons migrating in the cortex.

To complement our analysis of endogenous PCDH19 production, we generated two mouse lines: a C-terminus-tagged PCDH19-V5 mouse line, which would allow detection of PCDH19 with higher sensitivity, as the V5 antibody has been verified in more studies; a PCDH19 KO mouse line, which served as a negative control for the HCR analysis (Supplementary Figures 1B,C). The PCDH19 KO mouse line was designed to have a large deletion of 1,186 bp in the first exon 1. This deletion leads to a frameshift from AA176 onwards and a truncation of the protein at AA189. We could not observe any HCR signal nor any protein production in this knockout model (Supplementary Figures 1C,D), also not on any other height, ruling out alternative start exons that might generate a residual intracellular domain. Western Blot analysis of brain lysates showed the production of PCDH19-V5 with a stronger band around 126 kDA at E13.5, E14.5, and postnatal Day (P)7 (Supplementary Figure 1E). The multiple bands at the full-length size could represent different known PCDH19 mouse isoforms (Hunt et al., 2018) or post translational modifications. In the embryonic and P7 samples, but not in the adult brain, an additional band was detected at 50 kDA, the predicted MW of the PCDH19 intracellular domain. This fragment might have been generated by enzymatic cleavage, similar to some other PCDHs, as has been shown recently (Pancho et al., 2020; Gerosa et al., 2022). Besides a weak band around 70 kDa, no bands were observed in the wild-type control, showing the specificity of the V5 antibody. Our data thus suggested that, in the developing brain, PCDH19 appeared in different forms, including a cleaved version.

### Distinct subdomains target PCDH19 to different subcellular localizations

**Figure 2:**
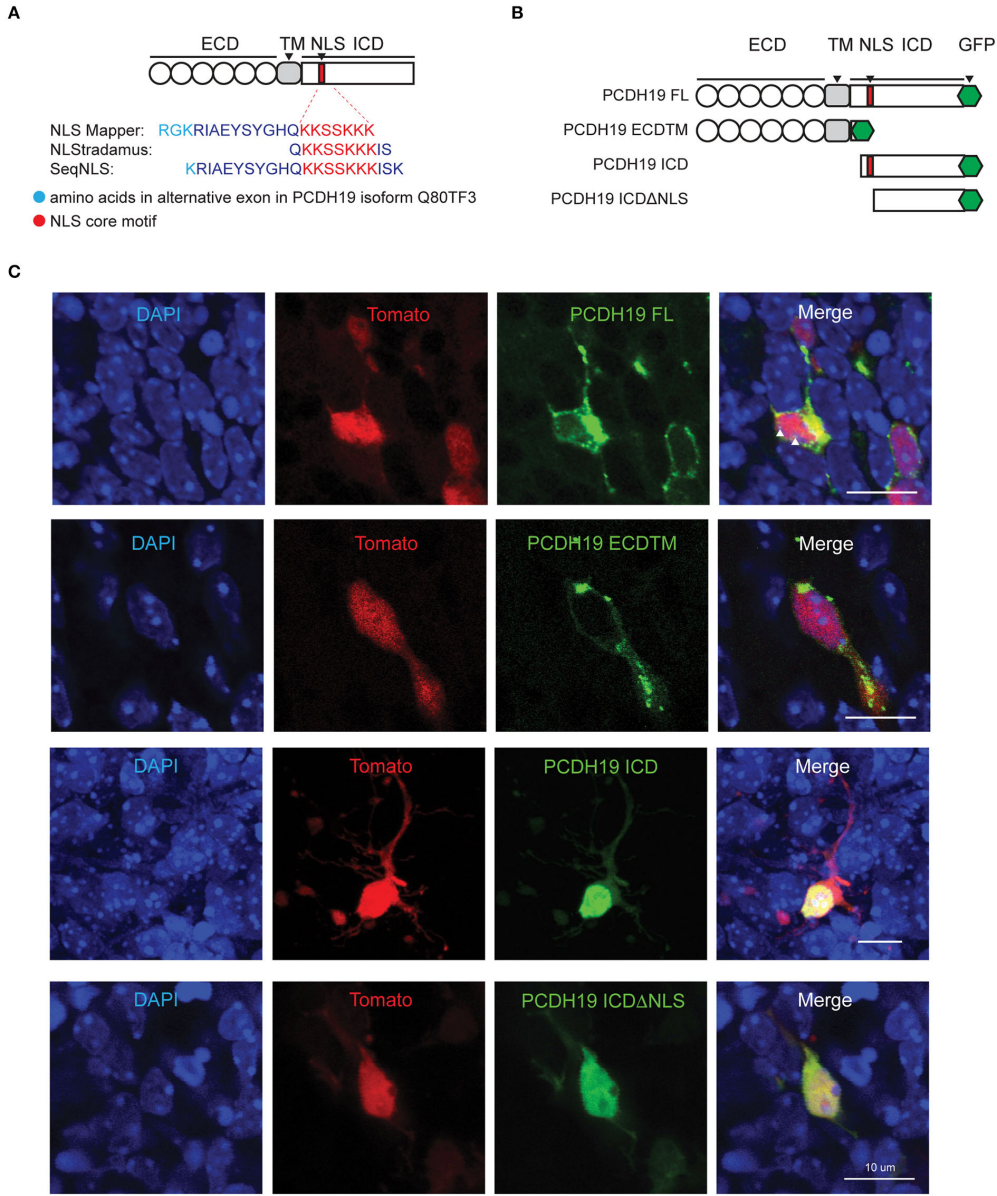
PCDH19 subdomain constructs target expression to different subcellular locations. **(A)** NLS prediction within the mouse PCDH19 amino acid sequence with three different programs. Cyan represents the alternate exon and red the consensus sequence. **(B)** Full-length PCDH19 and overexpression subdomain constructs were produced C-terminally tagged with a GFP tag and inserted into a bicistronic plasmid containing TdTomato. **(C)** PCDH19 subdomain expression in slice EP neurons 72 h after electroporation. Separate fluorescence channels, as well as the overlay, show a similar subcellular localization to the Neuro2A cells. White arrowheads in PCDH19 FL indicate a GFP signal in the nucleoli.

Cleavage of the ICD of transmembrane proteins is often followed by nuclear translocation, driven by a nuclear localization signal. We used distinct prediction tools to search for nuclear localization signals within the PCDH19 sequence (Figure 2A). Three NLS sequences of the longest PCDH19 isoform (Q80TF3) were predicted, yet scored differently in the prediction program NLStradamus (Supplementary Figure 2A) (Nguyen Ba et al., 2009).

To further study the subcellular localization of distinct PCDH19 subdomains, we generated C-terminally tagged overexpression constructs of subdomains of PCDH19 in bicistronic TdTomato expression plasmids (pCAGGS-PCDH19-eGFP-IRES-TdTomato): a full-length version: PCDH19FL; extracellular and transmembrane domains: PCDH19ECDTM, an intracellular domain: PCDH19ICD and intracellular domains without a predicted nuclear localization signal (NLS): PCDH19ICDΔNLS (Figure 2B). PCDH19ICDΔNLS was lacking the predicted NLS with the highest score and analysis with NLStradamus showed loss of most nuclear localization activity (Supplementary Figure 2A). The constructs were validated by expressing them in Neuro 2a cells (Supplementary Figure 2C). Western Blot analysis indicated a proper production of the GFP-tagged domain mutants (Supplementary Figure 2B). PCDH19 FL overexpression resulted in accumulation of tagged PCDH19 around the nuclear membrane. Overexpression of PCDH19ECDTM resulted in accumulation of PCDH19 at the membrane of the cell. PCDH19ICD localized to the nucleus, while PCDH19ICDΔNLS could be found distributed over the whole cell and less prominent in the nucleus. Therefore, removing the NLS in this construct seems to inhibit nuclear translocation at least partially. Indeed, when we quantified the nuclear-cytoplasmic ratio (Supplementary Figure 2D), we found that PCDH19ICD localized preferentially in the nucleus, while this preference was lost upon removal of the NLS. To investigate whether overexpression in the developing mouse forebrain yields similar results, we performed slice electroporation and visualized subcellular distribution of GFP-tagged PCDH19. A similar subcellular distribution could be observed: PCDH19ECDTM was mainly found at the membrane. PCDH19FL was at the membrane, strongly again surrounding the nucleus but could also be identified in the nucleoli. PCDH19ICD localized to the nucleus while PCDH19ICDΔNLS was distributed all over the cell (Figure 2C). Taken together, our data show that PCDH19ICD translocated to the nucleus, probably using the predicted NLS. In addition, these constructs now allowed us to study the role of PCDH19 at the membrane and distinguish it from a potential role in the nucleus in different mosaic overexpression paradigms.

### Cortical interneuron migration is decreased by gain-of-function of *Pcdh19*

**Figure 3:**
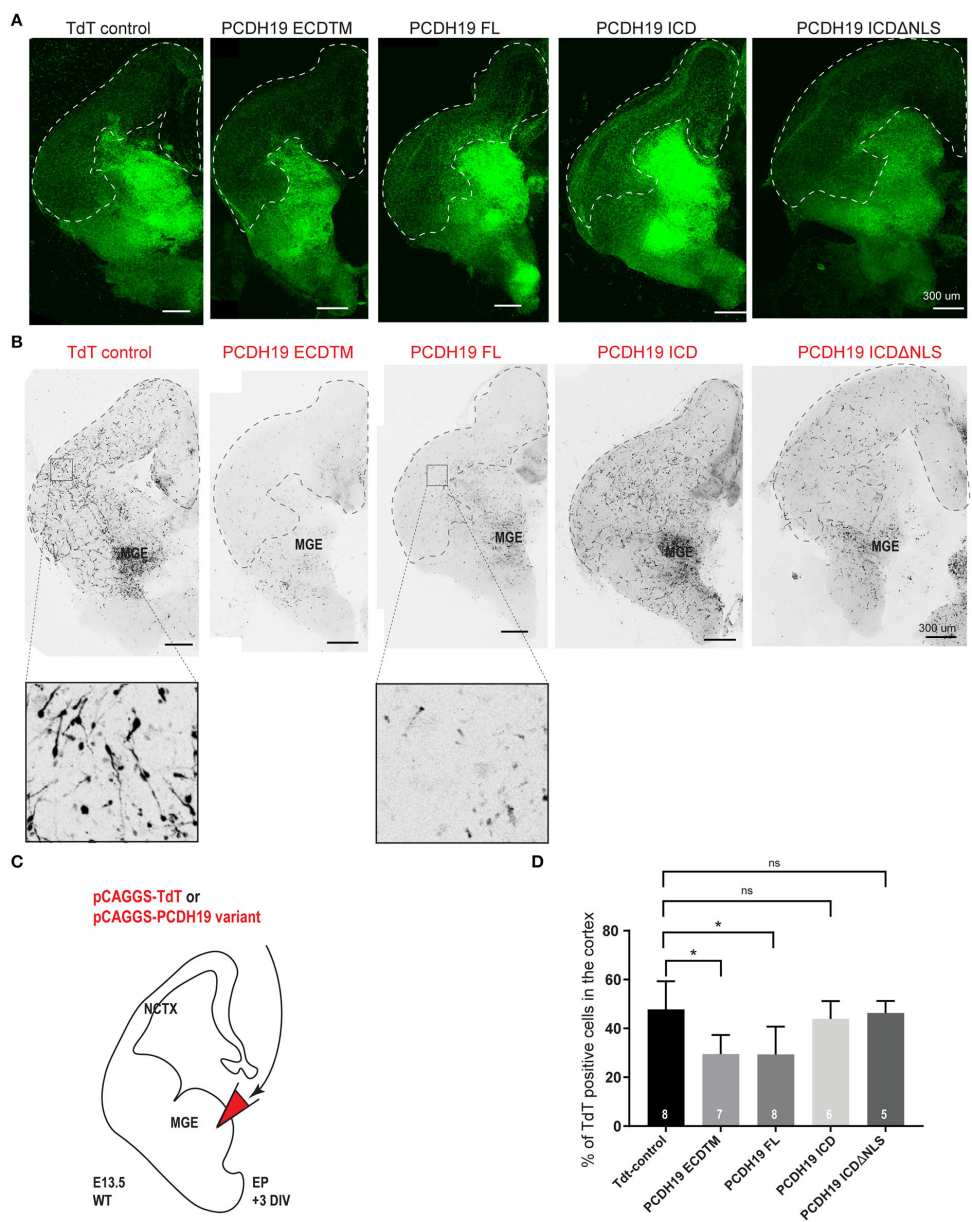
PCDH19 extracellular domain misexpression significantly decreases cortical IN migration. **(A)** 72-h post electroporation taken Z-stack example images of brain slices of TdT control and various PCDH19 overexpression constructs for the Dlx5/6-CRE-IRES-eGFP fluorescence, including the dotted line marking the cortical field. **(B)** Z-stack example images of the brain slices shown in A of TdT control and diverse PCDH19 overexpression constructs for the electroporated neurons (Tomato fluorescence); the dotted line shows the cortical field. **(C)** A schematic of the *ex vivo* brain slice electroporation setup to study the overdose of PCDH19 on cortical IN migration from MGE-derived IN. Analysis of 72-h post electroporation in Dlx5/6-CRE-IRES-eGFP brain slices electroporated with bicistronic plasmids co-expressing a PCDH19 subdomain and TdTomato or only TdTomato in the empty bicistronic plasmid that was used as control. **(D)** After 72 h in culture, IN migration to the cortical field was determined by measuring the intensity of the TdT^+^ neurons within the cortical field divided by the intensity of the TdT^+^ neurons spread over the whole brain slice. Upon PCDH19 ECD TM misexpression, significant reduction in cortical IN migration to the cortical field could be measured [the Kruskal–Wallis multicomparison test with the Dunn's *post hoc* test, *p = 0.0312 (PCDH19 ECDTM) and *p = 0.0122 (PCDH19 FL)]. N number of replicates is indicated within the bars.

As PCDH19-CE only affects women that display cellular mosaicism of PCDH19, the imbalance in PCDH19 dosage at cell-cell contact sites might be a driver of the phenotype. We decided to model this mosaic imbalance by altering PCDH19 levels in some cells only by electroporation. In order to follow up the survival and quality of the slices, we used Dlx5/6-Cre-IRES-eGFP organotypic brain slices in which proper interneuron migration of non-targeted cells was a proxy for healthy slices. A PCDH19 subdomain-expressing plasmid or the empty TdTomato control plasmid was electroporated in the MGE. This allowed us to study the effect of overdosing distinct domains of PCDH19 on the migrational behavior of MGE cortical IN after 3 days in organotypic culture (Figures 3A–C). Representative images of organotypic brain slices for the endogenous fluorescence Dlx5/6ireseGFP (Figure 3A) and the electroporated fluorescence TdTomato (Figure 3B) after 3DIV are shown for all conditions, and the cortical field is depicted by the dotted line (Figures 3A,B). Quantification of the percentage of TdT positive neurons within the cortical field compared to the whole brain slice showed significantly reduced cortical IN migration for PCDH19ECDTM and PCDH19FL compared to the control condition (Figure 3D, Kruskal–Wallis test with the Dunn's *post hoc* test). Stunted neuronal morphology could be observed after PCDH19FL (Figure 3B) as well as after PCDH19 ECDTM overexpression, suggesting that increasing the PCDH19 extracellular amount could affect cell morphology or survival. In contrast, increasing the dosage of the intracellular domain of PCDH19 did not impact IN migration nor morphology. As we hypothesized that an imbalance in PCDH19 dosage between migrating interneurons and their environment would influence migration, one could argue that non-targeted migrating interneurons might be affected as well when neighboring, co-migrating interneurons would express an excessive amount of PCDH19. We therefore investigated the migration of all eGFP-labeled neurons, and although we saw a reduction, this effect was not significant (Supplementary Figure 3, Kruskal–Wallis test), potentially because only a fraction of interneurons was targeted. Our data thus indicated that overdosing in especially the extracellular part of PCDH19 hampered tangential migration of interneurons to the neocortex.

### *Pcdh19* gain-of-function affects migration distance and affects neuronal processes

**Figure 4:**
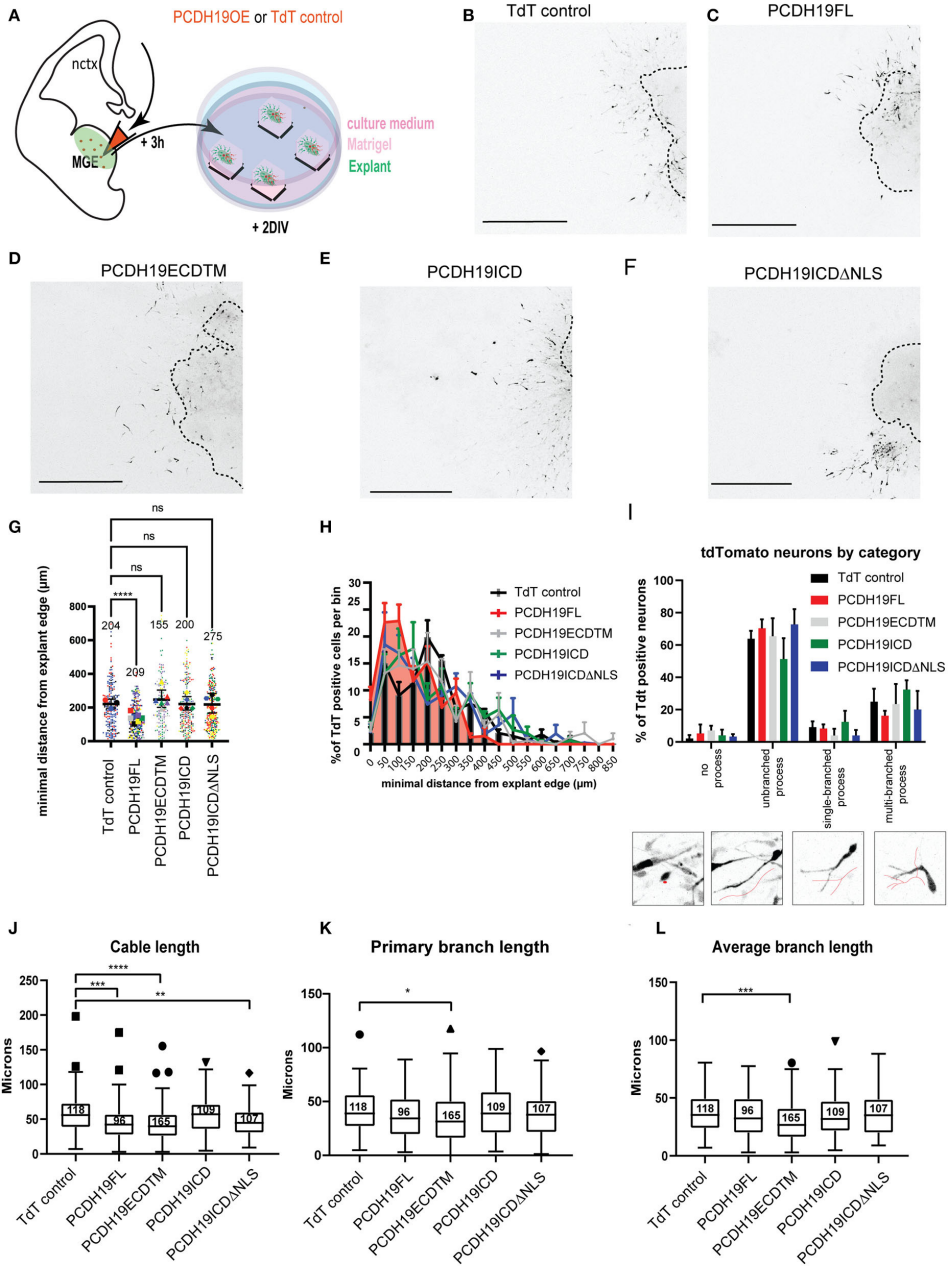
Effects of overexpressing PCDH19 subdomains on MGE cell migration and morphology. **(A)** A schematic of the *ex vivo* MGE explant electroporation setup to investigate the effect of PCDH19 overexpression in MGE-derived IN. Total minimum migration distance ‘d' was assessed after 48 h of electroporation in Dlx5/6-CRE-IRES-eGFP MGE explants with a PCDH19 construct or the empty TdTomato plasmid. **(B–F)** Example images of electroporated and cultured explants 48 h post electroporation of the TdTomato control plasmid **(B)** and PCDH19 constructs **(C–F)**. **(G)** A dot plot depicting tdTomato^+^ IN-related minimal distance from the explant edge. Each dot represents one electroporated neuron in the respective condition; colors of the dots relate to different explants. Significantly shorter distance from the explant edge could be measured between PCDH19 FL and TdT control (the Kruskal–Wallis non-parametric test and the Dunn's *post hoc* test, ^****^p < 0.0001). **(H)** Quantification of TdTomato neurons per bin normalized against the total amount of TdT neurons per bin showed non-significant difference per bin (the mixed model ANOVA test). **(I)** A bar chart showing the percentages of INs that were classified into 4 different categories (insets depicting the diverse neuron morphology categories) from different electroporated explants calculated on the total amount of INs per category within each experimental group. Nonsignificant differences could be identified within the experimental groups per category (two-way repeated measures ANOVA with Holm-Sidak *post hoc* comparison). **(J–L)** Boxplots depicting TdTomato^+^ neuron-associated cable length **(J)**, primary branch length **(K)**, and average branch length **(L)** measurements in TdT control and PCDH19 constructs-electroporated MGE explants. Significant differences could be measured in the neuron cable length between PCDH19 FL and TdT control (^****^p < 0.0001), PCDH19 ECDTM, and TdTcontrol (^****^p < 0.0001) and PCDH19ICDΔNLS and TdT control (^*^p = 0.0026) (the Kruskal–Wallis non-parametric test and the Dunn's *post hoc* test). The primary branch length (the Kruskal–Wallis non parametric test and the Dunn's *post hoc* test, ^*^p = 0.0118) and the average branch length (the Kruskal–Wallis non parametric test and the Dunn's *post hoc* test, ^***^p = 0.0003) were significantly shorter in PCDH19 ECDTM compared to TdT control. DIV, days in vitro; scale bar: 500 μm.

To further study the effect of excess PCDH19 on neuronal migration and morphology, we investigated PCDH19 overexpressing neurons in cultured MGE explants. Interneurons generated from these explants migrate out over the course of hours, during which they detach from the explant and make plenty short-term contacts with migrating cells in their vicinity. Besides revealing the impact of PCDH19 gain-of-function on individual cells and their migration capacity, this assay can also uncover disturbed cell-cell adhesion. E13.5 Dlx5/6-Cre-IRES-eGFP MGE explants were electroporated with the different PCDH19 subdomain expressing and control constructs, allowing the detection of electroporated (GFP + TdT +) vs. non-electroporated (GFP + TdT–) cells (Figure 4A). Next, we measured the total migration distance after 2DIV. Example images are shown for explant per experimental condition (Figures 4B–F). Statistical analysis with the Kruskal–Wallis non-parametric test and the Dunn's *post hoc* test showed a significant reduction in the spread of PCDH19FL-expressing Ins compared to the TdT control condition (*p* < 0.0001) (Figure 4G). Binned distribution analysis indicated that PCDH19FL-overexpressing IN distribution is closer to the explant than the rest of the conditions; nevertheless, no particular bin-specific means were found to be significantly changed (mixed-model ANOVA) (Figure 4H).

Similarly, as in the brain slice, we asked whether we would see a non-cell autonomous effect and, therefore, also analyzed non-targeted eGFP-positive neurons (Supplementary Figure 4). Also here, statistical analysis with the Kruskal–Wallis non-parametric test and the Dunn's *post hoc* test displayed a significant reduction in the spread of eGFP + TdT – neurons in the PCDH19FL condition (*p* < 0.0001) compared to the TdT control, and, unexpectedly, a significantly larger spread of the eGFP + TdT – neurons in the PCDH19ICD condition (*p* < 0.0001) (Supplementary Figure 4F). Statistical analysis per bin was also not significant for the non-cell autonomous effect of PCDH19 overexpression (Supplementary Figure 4G).

To further address the previous observation of the stunted neuronal morphology in the brain slices, we wondered whether upon overexpression of the different subdomains neuronal morphology was affected. Each neuron was assessed depending on the morphology of the leading process into “no process”, “unbranched process”, “single-branch process”, and into “multi-branch process”, and then the percentage of total neurons per experimental condition per explant was calculated per phenotype (Figure 4I). Most neurons had an unbranched process (50–70%), which fits to the previous observed morphology in the explant assay (Mitsogiannis et al., 2021). Overexpression of PCDH19FL compared to the control TdT-control did not result in any significant observation. Since total migration distance was reduced upon PCDH19FL overexpression, we also decided to look into the topological morphological characteristics of the neuronal processes. Assessment of the total cable length, a measure for neurite outgrowth, resulted in significant differences for PCDH19FL, PCDH19ECDTM, as well as PCDH19ICDΔNLS, compared to the control condition (Figure 4J, the Kruskal–Wallis non-parametric test, and the Dunn's *post hoc* test). Primary branch length and average branch length were only significantly affected upon overexpression of PCDH19ECDTM compared to the control condition (Figures 4K,L, the Kruskal–Wallis non parametric test, and the Dunn's *post hoc* test). Thus, the observed reduced migration distance upon PCDH19FL overexpression cannot be explained only by an affected neuronal morphology.

### Overexpressing the extracellular part of PCDH19 increases apoptosis

**Figure 5:**
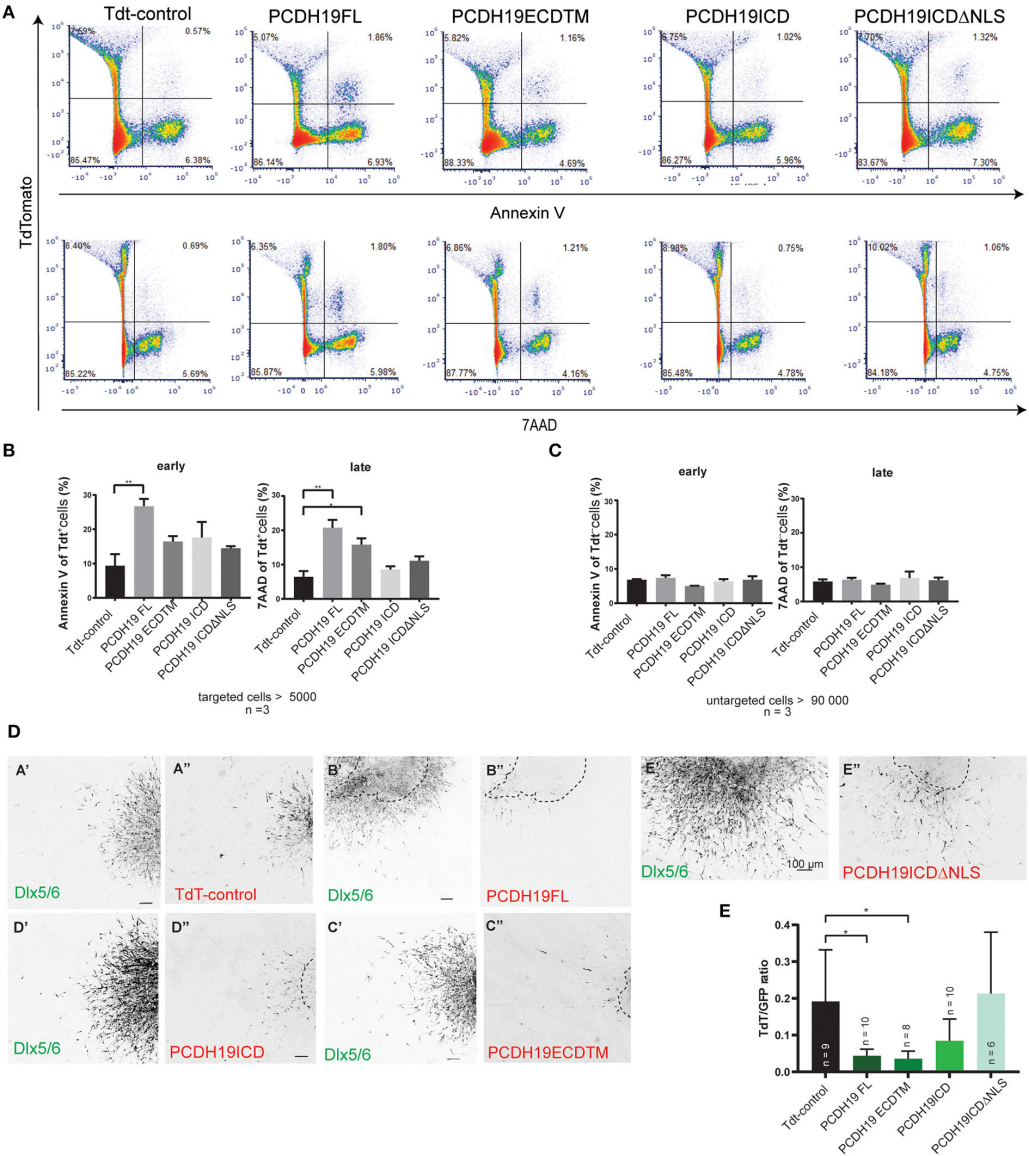
PCDH19 ECD overexpression increases apoptosis in Neuro 2A-transfected cells and might induce apoptosis in MGE electroporated INs. **(A)** Examples of flow cytometry pseudo color plots after 48 h of transfection of control and PCDH19 constructs. Early apoptotic detection is shown by AnnexinV fluorescence [**(A)**, the upper row], and late apoptotic detection is shown by 7AAD fluorescence [**(A)**, the lower row]. **(B)** Quantification of early apoptotic targeted cells indicated significant increase in PCDH19 FL compared to TdT control (the right panel, Welch ANOVA, and the Dunnet's *post hoc* test, ^**^p = 0.0025). Similarly, quantification of late apoptotic cells yielded significant increases in PCDH19 FL, PCDH19 ECD TM compared to Tdt control [the left panel, Welch ANOVA, and the Dunnet's *post hoc* test, ^**^p = 0.0038 (PCDH19 FL) ^*^p = 0.0112 (PCDH19 ECDTM)]. **(C)** Untargeted cells showed no significant differences in early nor in late apoptotic cells. **(D)** Explant examples of the control plasmid and the diverse PCDH19 constructs depicted in the GFP channel (Dlx 5/6) and in the red channel (TdT, PCDH19-eGFP-IRES-TdT constructs). Areas and shapes differ within the different experimental conditions; the original explant boundary is indicated by the dotted line. **(E)** Quantification of TdTomato intensity (a proxy for amount of targeted cells) relative to the GFP signal (Dlx5/6 INs) and normalized to the size of an explant. Significant less TdTomato-expressing cells are found upon overexpression of PCDH19 FL and PCDH19 ECDTM [the Kruskal–Wallis non parametric test and the Dunn's *post hoc* test, ^*^p = 0.0229 (PCDH19 FL) and ^*^p = 0.0219 (PCDH19 ECDTM)].

The observed impact on cortical IN migration and total migration distance could not be explained by aberrant cell morphology of the electroporated neurons only. Therefore, we sought to investigate whether cell survival was affected. First, we tested our hypothesis in the Neuro2A neuronal cell line. After 48 h of transfection with the PCDH19 or control constructs, apoptosis was measured using flow cytometry. AnnexinV was used to detect early apoptotic cells and 7AAD to detect late apoptotic cells within transfected and untransfected cells after overexpression. Flow cytometry example plots show transfected cells (TdT +) and early apoptotic cells (Figure 5A, the upper row) and late apoptotic cells (Figure 5A, the bottom row). Quantification of targeted cells results in more early and late apoptotic cells upon PCDH19FL overexpression compared to the control condition (Figure 5B, the left panel, Welch ANOVA, and the Dunnet's *post hoc* test) and in more late apoptotic cells upon PCDH19ECDTM and PCDH19FL compared to the control condition (Figure 5B, the right panel, Welch ANOVA, and the Dunnet's *post hoc* test). Quantification of early and late apoptotic cells in the untargeted cells resulted in non-significant differences, suggesting that the cells were not suffering because of the procedure, or that apoptosis was also induced by a non-cell-autonomous effect (Figure 5C, left and right panels). Therefore, the observed effect is specific for the PCDH19 overexpressing cell population, but only when the extracellular domain is present.

Next, we wondered whether we could address apoptosis in a more physiological model like the MGE explant. More cells can be targeted than in the slice electroporation with this approach, and, also, cellular resolution is better achieved with the assay than with the slice electroporation. We approximated the cell number of targeted cells *via* the TdT signal, again using the background of the Dlx5/6-Cre-IRES-eGFP mouse line. We reasoned that, if overexpression would affect cell viability and survival, we might lose more targeted cells and, hence, TdT expression, whereas overall GFP expression should stay constant. We measured the ratio between the TdT+ and GFP+ signals, whereby we normalized measurements by the explant area as shapes were diverse between measured conditions, and repeated the experiment several times to account for differences in electroporation efficiencies between experiments (Figure 5D). Our data consistently showed significantly lower ratios for PCDH19ECDTM and PCDH19FL compared to the control condition (Figure 5E, the Kruskal–Wallis non parametric test, and the Dunn's *post hoc* test), which could indicate that overexpression of these plasmids triggers neuronal loss. Taken together, our data suggest that an additional explanation for the observed impact on cortical interneuron migration could rely on increased neuronal cell death.

### *Pcdh19* loss-of-function affects migration distance and polarity of MGE-derived interneurons yet does not affect neuronal processes

Another way to model mosaic imbalance in PCDH19 dosage at cell-cell contact sites is to deplete PCDH19 levels in some cells only. We applied a CRISPR RNP electroporation approach to downregulate PCDH19. Two guides targeting Exon 1 were designed (P1, P2) (Supplementary Figure 5A) and validated *in vitro* on a PCDH19FLeGFPiresTdT construct expressed in Neuro2A cells. Both guides were able to significantly reduce the percentage of GFP-positive cells on the total number of transfected cells (Supplementary Figures 5B,C).

**Figure 6:**
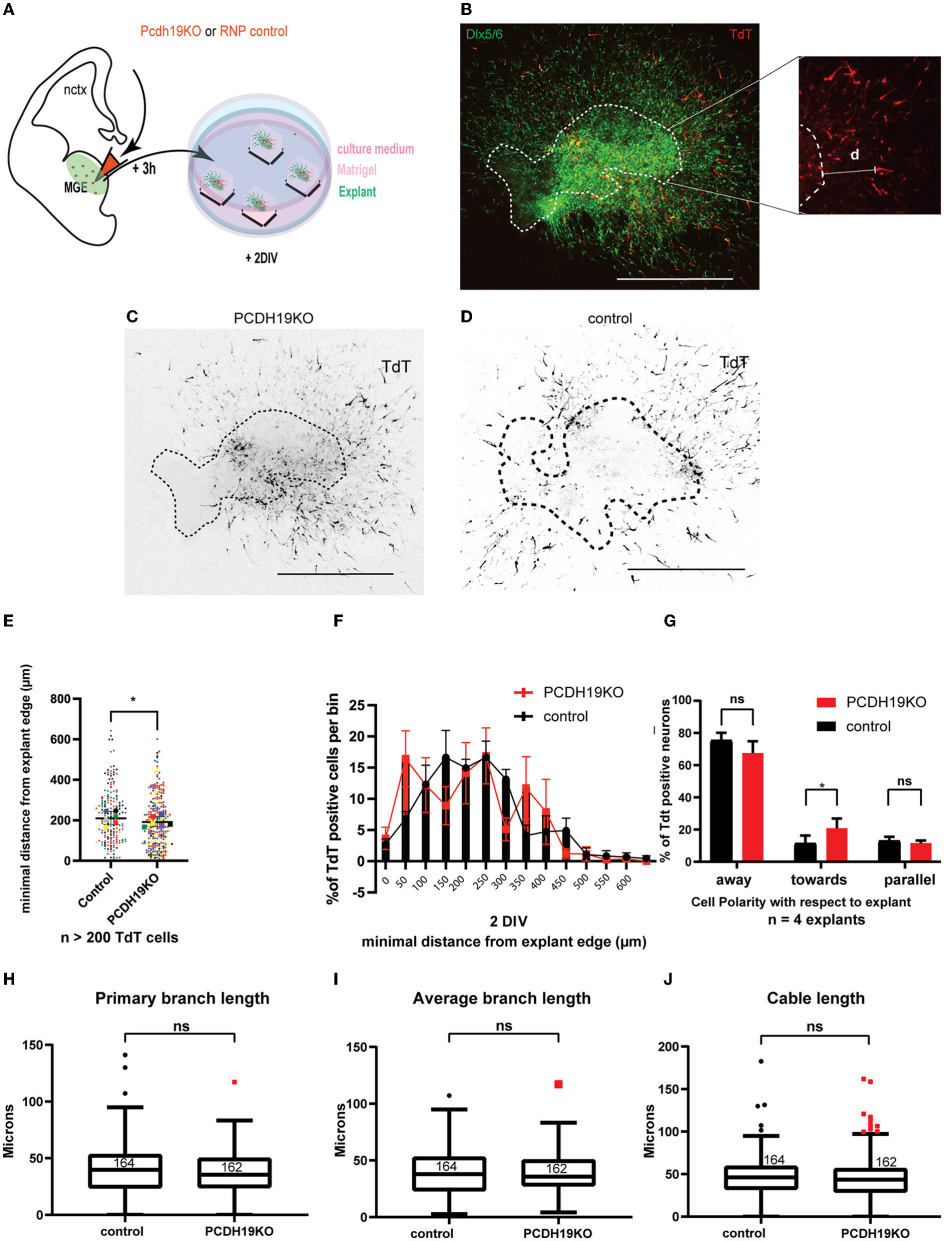
Subtle effect of PCDH19 loss on the total minimum migration distance of MGE explants-derived INs might arise from disturbed migration polarity. **(A)** A schematic of the *ex vivo* MGE explant electroporation setup to investigate the effect of PCDH19 KO in MGE-derived IN. **(B)** Total minimum migration distance “d” was assessed after 48 h of electroporation in Dlx5/6-CRE-IRES-eGFP MGE explants with PCDH19KO RNPs co-electroporated with tdTomato. Minimum distance capacity was analyzed in electroporated neurons (red) measuring ‘d' in MGE-derived IN (green). **(C,D)** Representative images of electroporated and cultured explants 48 h post electroporation of PCDH19KO RNP **(C)** and control Cas9 **(D)**. **(E)** A dot plot depicting tdTomato^+^ IN-related minimal distance from the explant edge. Each dot represents one electroporated neuron in the respective condition; colors of the dots relate to different explants. Significantly shorter distance from the explant edge could be measured between PCDH19KO and control (the Mann–Whitney *U* test, ^*^p < 0.05). **(F)** Quantification of TdTomato neurons per bin normalized against the total amount of TdT neurons per bin showed non-significant difference per bin (the Mann–Whitney *U* test, followed by multiple false discovery rate corrections). **(G)** Polarity with respect to the explant of more than 120 TdTomato neurons was assessed in 4 explants, showing significantly more neurons migrating toward the explant in the PCDH19KO experimental condition. (Two-way repeated measures ANOVA with Holm-Sidak *post hoc* comparison, ^*^p < 0.05). **(H–J)** Boxplots depicting TdTomato^+^ neuron-associated primary branch length **(H)**, average branch length **(I)**, and cable length **(J)** measurements in control and PCDH19KO conditions. No significant differences could be detected in these morphology-associated aspects. DIV, days *in vitro*; IN, interneuron; RNP, ribonucleotide protein; ns, not significant; scale bar: 500 μm.

To address migration characteristics at the cellular level, we again used MGE tissue derived from Dlx5/6-Cre-IRES-eGFP mice. Embryonic brains were injected and electroporated with ribonuclear particles containing a guide targeting *Pcdh19* and Cas9 protein (referred to as PCDH19KO RNPs) or Cas9 protein alone (control), and a TdT-expressing plasmid in the MGE at E13.5. Subsequently, MGE pieces were cultured in Matrigel for 2 days (DIV) (Figure 6A). At 2 DIV, total migration distance was measured from the edge to the neuronal soma (Figure 6B). Figures 6C,D show representative examples of TdT-labeled neurons for each condition images. Migration distance was measured for more than 200 neurons in the PCDH19KO and control condition. Statistical comparison with the Mann–Whitney U test resulted in a slight significant decrease (*p* < 0.05) of total migration distance upon loss of function of *Pcdh19* (Figure 6E). Binned distribution analysis of the migration distance suggested that, in the PCDH19KO condition, more neurons are distributed closer to the explant; however, no bin-specific means were found to significantly differ (Multiple Mann–Whitney tests with FDR for multiple corrections) (Figure 6F). Also, for this analysis, we addressed the non-cell autonomous effect of the PCDH19KO condition on eGFP+TdT– neurons and detected a significant decrease (*p* < 0.001) compared to the control condition (the Mann–Whitney *U* test) (Supplementary Figure 6). We further investigated whether the direction of migration could be affected in the PCDH19KO-electroporated neurons. To this aim, we classified the leading neurite direction, showing away from the explant, toward the explant or when the direction could not be specified in both categories, parallel to the radial migration. The total amount of neurons within these three classifications was counted in 4 explants for each experimental condition. Statistical analysis indicated that more leading neurites were pointed toward the explant in the PCDH19KO condition (two-way repeated measures ANOVA with Holm–Sidak *post hoc* comparison, *p* < 0.05) (Figure 6G). Overall, about 70% of the counted neurons pointed their main neurite away from the explant in both conditions. During interneuron migration, extension of the leading process is followed by branching as the neuron senses the environment, and, eventually, nucleokinesis. Microtubule and actin cytoskeleton remodeling events govern these dynamic processes (Bellion et al., 2005; Métin et al., 2006; Guo and Anton, 2014). A second reason for hampered migration could be disturbed by branch elongation and secondary branch formation. Hence, we measured primary branch length (Figure 6H), average branch length (Figure 6I), and cable length (Figure 6J) for more than 200 neurons in each condition, yielding no difference between the conditions.

In addition, we also assessed cell survival in a similar manner as described above (Figure 5D). Reducing the level of PCDH19 did not affect cell survival; we observed a lower number of targeted cells only in the control condition; however, this difference remained statistically non-significant (the Mann–Whitney *U* test) (Supplementary Figure 7).

Collectively, these results indicated that, similar to PCDH19 overexpression, loss of PCDH19 in MGE-derived IN mildly reduced the total migration distance in MGE explants, which might be explained in part by a disturbed migration polarity. However, the cell-cell variation in the effectiveness of the knockout, as well as the potential off-target effects, was difficult to measure in this explant model.

### Mild reduction in cortical interneuron migration in an *in vivo* model of PCDH19-CE

**Figure 7:**
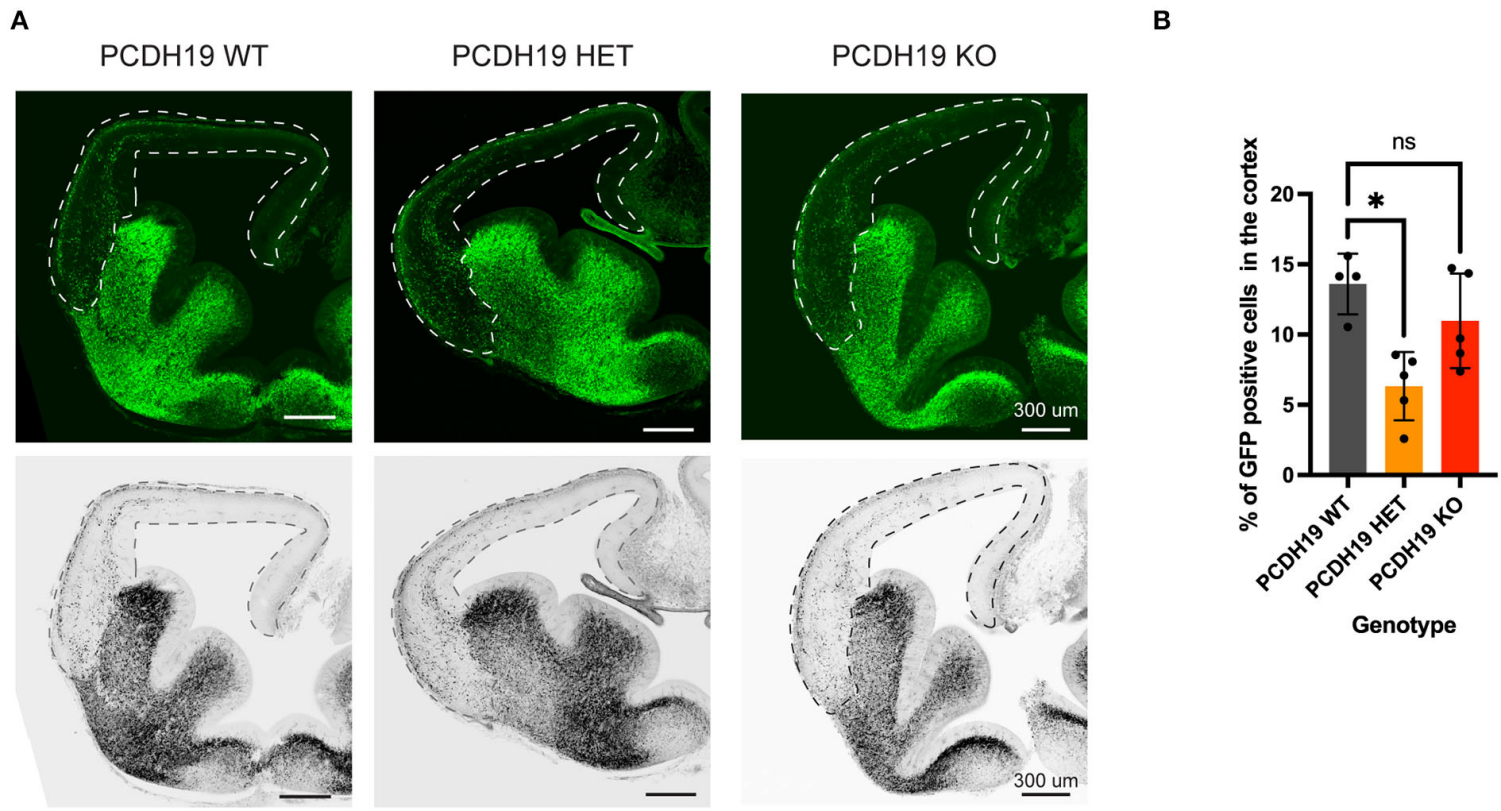
Cortical interneuron migration upon loss of PCDH19 *in vivo*. **(A)** Representative brain slices for the PCDH19 WT mouse, the PCDH19 HET mouse, and the PCDH19 KO mouse; the dotted line represents the area that was measured. **(B)** Quantification of GFP positive cells in the measured area shows average of GFP-positive cells percentage in the cortex per mouse genotype. Less migration to the cortex could be detected in the PCDH19 HET mouse compared to the control PCDH19 WT (*p = 0.0135), the Kruskal–Wallis test, and the Dunn's *post hoc* test.

To further study the impact of PCDH19 imbalance and its impact on cortical interneuron migration, we modeled the mosaic loss by crossing the PCDH19 knockout mouse line heterozygotes with Dlx5/6-Cre-IRES-eGFP mice in order to obtain control (PCDH19+/+; Dlx5/6-Cre-IRES-eGFP), heterozygotes (PCDH19+/–; Dlx5/6-Cre-IRES-eGFP), and PCDH19 KO (PCDH19–/–; Dlx5/6-Cre-IRES-eGFP) mice in which VT-derived interneurons would be eGFP labeled. We studied the proportion of labeled cells in the cortex at E13.5 as a proxy for cell migration, similar to the analysis performed on the slices (Figure 7A). In doing so, we found that, at this early stage, we could detect a mild but significant reduction in migration in the heterozygote mice, but not in the homozygous KOs [Figure 7B, (^*^p = 0.0135, the Kruskal–Wallis test, and the Dunn's *post hoc* test)]. These data suggest that, also, *in vivo*, creating an imbalance in the dosages of PCDH19 in interneurons and neurons hampers the overall interneuron migration to the cortex.

## Discussion

This paper investigated the hypothesis that the early-onset epilepsy in females bearing a loss-of-function mutation on one allele of the *PCDH19* gene might be linked to a developmental failure of pallial interneurons to properly migrate to the pallium. Our study shows presence of *PCDH19* expression in the developing forebrain, including those regions generating pallial interneurons, and, in particular, demonstrated a temporally and spatially dynamic pattern. At the level of individual interneurons, we could detect variation in expression levels between cells, suggesting that different cell types might depend on PCDH19 to a different extent. This observation was done at the RNA level, so at this point, it is still unknown whether this translates to effectively variable amounts of PCDH19 at the membrane level. The level of PCDH19 differs between cells, regions, and time points, which suggests that expression of this gene is tightly regulated. Specific types of interneurons might thus be depending on PCDH19 at different stages of their development. This corroborates the findings that PCDH19 has been implicated in neurogenesis (Cooper et al., 2015; Fujitani et al., 2017; Homan et al., 2018; Lv et al., 2019), neural sorting (Pederick et al., 2018), and synaptogenesis and function (Bassani et al., 2018; Serratto et al., 2020; Hoshina et al., 2021; Mincheva-Tasheva et al., 2021). Our study focused on a, hitherto, less well-studied role in neural migration, taking the role of PCDH19 subdomains into account.

We could not detect an increase in neuronal migration in our loss of function studies in contrast to the previous *in vitro* observation for cortical PCDH19KO neurons (Pederick et al., 2016). This discrepancy might arise from the diversity of the assays, from the cell type investigated, as well as from the context of the experiment. Our analysis measured the total migration distance of PCDH19KO cortical interneurons that were still mixed with PCDH19 WT cortical interneurons, while Pederick et al. (2016) made their observation using full KO neurospheres. Our binning analysis together with the polarity assessment detected neurons migrating toward the explant, suggesting a disturbed migration polarity. Along the same line, ectopic positioning and orientation of PCDH19 loss of function hippocampal neurons have been detected in rats (Bassani et al., 2018). In human iPSC progenitors, PCDH19 is found in a polarized manner at the apical membrane, suggesting a role in polarity (Compagnucci et al., 2015). Whether PCDH19 loss affects neuronal polarity and if these could contribute to the phenotype of PCDH19-CE remains to be investigated.

Our data suggest that, in the developing mouse brain, PCDH19 is proteolytically processed, and the cytoplasmic fragment accumulates in the nucleus. Protein processing has been described for classical Cadherins (Marambaud et al., 2002; Maretzky et al., 2005; Reiss et al., 2005; Symowicz et al., 2007; Jang et al., 2011; Conant et al., 2017) and, for other Protocadherins, such as PCDH12 (Bouillot et al., 2011) and the clustered α- and γ-PCDHs (Junghans et al., 2005; Reiss et al., 2006; Buchanan et al., 2010). The released cytoplasmic fragment of Cadherins is bound by CREB-binding protein and diverged to the proteasome, suggesting that processing serves a role to remove the adhesive contact. The cytoplasmic fragment of PCDHγC3 that is released travels to the nucleus and might exert a role there (Haas et al., 2005). Recently, PCDH19 has been shown to be proteolytically processed in an activity-dependent manner in rat hippocampal cells and human iPSC-derived neurons, further confirming our findings (Gerosa et al., 2022). Upon overexpression of full-length PCDH19 in Neuro2A cells, the majority of the protein appeared in the cytoplasm and on the membrane, and not in the nucleus. This might suggest that either the necessary processing machinery is not present in Neuro2A cells, and/or additional conditions need to be fulfilled to elicit cleavage. In our *ex vivo* slice electroporation neurons, we did detect a signal in the nucleoli; a similar localization was obtained in hippocampal neurons for PCDH19FL, and the cytoplasmic fragment of PCDH19 also localized to the nucleus in this study (Pham et al., 2017). Our data indicate that proteolytic processing is also happening *in vivo*, during the embryonic period, which suggests that it might play a more general role, beyond the synaptic role suggested by activity-dependent processing in mature neuronal cultures.

We applied different overexpression and knockout paradigms to mimic the mosaic imbalance of the disease condition during embryonic forebrain development, and found that, in general, having an excessive amount of PCDH19 (or facing absence of PCDH19 on neighboring cells) leads to defects in migration. This seemed to originate from the extracellular domain, as overexpression of the intracellular domain had no such effect, and was, at least, partly caused by enhanced apoptosis or reduced cell survival. We, therefore, hypothesize that having an excessive level of unbound PCDH19 at the membrane might trigger an apoptotic response.

A similar concept has been described for dependence receptors (Fombonne et al., 2012; Genevois et al., 2013; Causeret et al., 2016). These are a group of membrane proteins that need to be bound by a ligand in order for the cell to survive, or, conversely, when unbound, will trigger an apoptotic response (Mehlen and Bredesen, 2004). It remains to be investigated whether PCDH19 fulfills the criteria of a dependence receptor; whether it can be cleaved by caspases and initiate apoptosis, or whether it acts indirectly by stabilizing a known dependence receptor at the membrane. Interesting is the finding that PCDH19 interacts with Protein Tyrosine Phosphatase Non-Receptor Type 13 (PTPN13), a protein that prevents apoptosis when bound to NGFR, a known dependence receptor (Emond et al., 2020). Stabilization of a dependence receptor also fits with the observation that a truncated PCDH19-ECD, which is unlikely to exert any cytoplasmic interactions, can trigger apoptosis upon overexpression as well.

Other protocadherins have been linked to apoptosis in a different manner. Deletion of the whole γ-PCDH-cluster leads to a very particular loss of interneurons, namely, cortical interneurons (Carriere et al., 2020; Mancia Leon et al., 2020). Along the same line, removing PCDHγC4 from cortical or cerebellar interneurons results in significant losses in interneuron cell number, caused by apoptosis, suggesting that this isoform sustains interneuron survival (Garrett et al., 2019; Mancia Leon et al., 2020). Although these functions seem to be contradictory to the idea of dependence receptors, they do link PCDHs to the process of apoptosis. We also refer to our recent review, describing the importance of PCDH dosage control for neuronal survival in the brain (Pancho et al., 2020).

The fact that different PCDH19 domains induce opposite migration behaviors non-cell-autonomously is more difficult to explain. On the other hand, it indicates that cell-cell interactions occuring in these explants might be differrentially affected by extracellular or intracellular PCDH19 domains. The PCDH19 intracellular domain, which is translocated to the nucleus, might exert a nuclear role and change gene expression of immediate early genes (IEGs) as shown recently in hippocampal neurons (Gerosa et al., 2022). These IEGs perhaps initiate a gene expression cascade that results in the secretion of a factor that stimulates cell migration. The reduced migration of non-targeted cells upon overexpression of PCDH19FL but not PCDH19 ECD might be caused by a potential binding partner at the cytoplasmic domain, such as Cdh2 (N-Cad), which is a known a PCDH19-binding partner (Emond et al., 2011). During neurulation, both PCDH19 and Cdh2 need to be present to obtain directional and coherent migration of cells (Biswas et al., 2010). Cdh2 has been shown to be important for interneuron migration speed, polarity, and postnatal survival (Luccardini et al., 2013, 2015; László et al., 2020). Further research is needed to validate whether PCDH19FL overexpression increases the expression of Cdh2 at the surface and thus non-cell-autonomously might influence migration. For the moment, these remain speculations that need further experimental investigation; however, the influence of PCDH19-interacting partners is not to be neglected.

Although we found a decrease in interneurons populating the PCDH19 heterozygous cortex at a very early time point, other mouse models of the disorder could not demonstrate a significant cell loss in cortical interneurons at P20 or pyramidal neurons or a thinning of cortical layers at P10 (Galindo-Riera et al., 2021). In these models, wild-type and PCDH19 knockout cells reorganize themselves into columns of cells with similar genotypes, resulting in a decrease of imbalanced cell-cell contacts (Pederick et al., 2018). Combined with the cell-specific pattern of expression, a potentially hazardous imbalance situation might be avoided. On the other hand, the PCDH19 mosaicism might only delay the migration of interneurons, and the situation might be normalized at young postnatal stages.

Future studies on the endogenous interaction partners of PCDH19 in the developing brain will hopefully aid in revealing the pathways involved in the dynamic action of this protein in neurogenesis, neural survival, differentiation, migration, and synaptic function and plasticity.

## Data availability statement

The original contributions presented in the study are included in the article/Supplementary material, further inquiries can be directed to the corresponding author

## Ethics statement

The animal study was reviewed and approved by ECD Ethical Committee on Animal Experimentation, KU Leuven, Leuven, Belgium. The generation of the PCDH19-V5 tagged mouse and the PCDH19 KO mouse were done in accordance with European, national and institutional guidelines and approved by the State Office of North Rhine-Westphalia, Department of Nature, Environment and Consumer Protection (LANUV NRW, Germany; animal study protocol AZ 84-02.04.2014.A372).

## Author contributions

ES and AP conceived the research project and designed the experiments. AP performed all the experiments unless other mentioned and analyzed experimental results and data. MM helped in all MGE explant and brain slice electroporation experiments. TA designed the sgRNA P1 and P2 and helped in experiments. MD performed the analysis of the nucleo-cytoplasmic localization under supervision of PD. LE generated the sgRNA and ssODN and performed the experiments to generate the PCDH19-V5-tagged mouse and PCDH19 KO mouse under supervision of BS. PH did the NLS prediction. LE and KS supervised by BS and FV respectively, produced plasmid constructs for the subcloning of *PCDH19-eGFP* constructs. RV, LN, and LG provided technical support in genotyping and WB. AP and ES wrote the manuscript. All the authors contributed to the article and approved the submitted version.

## Funding

This work was supported by funding of the FWO (G0B5916N) and the KU Leuven 1051 (C14/16/049). BS was supported by the German Research Foundation (SCHE1562/8-1 and SFB1403, project No. 414786233). MD was supported by funding of the FWO (1S39418N).

## Conflict of interest

The authors declare that the research was conducted in the absence of any commercial or financial relationships that could be construed as a potential conflict of interest.

## Publisher's note

All claims expressed in this article are solely those of the authors and do not necessarily represent those of their affiliated organizations, or those of the publisher, the editors and the reviewers. Any product that may be evaluated in this article, or claim that may be made by its manufacturer, is not guaranteed or endorsed by the publisher.
