# Supplementary material for: Modifying PCDH19 levels affects cortical interneuron migration
Source: Front Neurosci. 2022 Oct 25;16:887478. doi: 10.3389/fnins.2022.887478 (PMC9642031; doi:10.3389/fnins.2022.887478)
Supplement: Supplementary file 1 [file Data_Sheet_1.pdf]

frontiers

Supplementary Material

1 Supplementary Figures and Tables

1.1 Supplementary Tables

Table S1: Primer sequences to genotype the Dlx5/6-Cre-IRES-eGFP.

cre	Sequence
Fwd	5'-TGCCACGACCAAGTGACAGCAATG-3'
Rev	5'-ACCAGAGACGGAAATCCATCGCTC-3'

Table S2 Primers to generate the PCDH19 PCR products. Target sequence for PCDH19 is underlined. Kozak sequence is shown in orange. HindIII restriction site (blue), MluI restriction site (green)

	PCDH19	Primer
Fwd	ICD	5'-CCCGCCAAGCTTGCCTGGATGGCAATCAAATGCAAGCGT-3'
	ICDΔNLS	5'-CCCGCCAAGCTTGCCTGGATGATTAGTAAGAATGACATC-3'
	ECDTM & FL	5'-GGGCCCGGTCTCAAGCTTATGGAGTCGCTCCTG-3'
Rev	ICDs	5'-CCCGCCACGCGTGAGAACGATATCCTTCAGACGCTT-3'
	FL	5'-GCGCGCGAGACCACGCGTGAGAACGATATCCTT-3'
	ECDTM	5'-GCGCGCGAGACCACGCGTTTTGATTGCCACGAA-3'

Table S3 Primer and ssODN sequence used to generate the Pcdh19-V5 mouse and KO

PCDH19			Sequence
KO	sgRNA	m6	5'-AGTGCCATTGATCAGAGGTGGGG-3'
		m7	5'-GGCCTGGAAATAAAGACGCGGGG-3'
		m8	5'-GGAGGCGACCCACCACACATGGG-3'
		m9	5'-CGACGTCAATGACAATACACCGG-3'
V5	sgRNA	Fwd	5'-TTAATACGACTCACTATAGGATCGTTCTCTAAAGCCATCTGTTTTAGAGCT AGAAATAGC-3'
		Rev	5'-AAAAAGCACCGACTCGGTGCC-3'
	ssODN		5'-AACCATTGGTGAGCAATTAAAATGAGCAGCTGCTACATCTTCACTAGCCAG TGTGGTTTCTGTTCTCTCTTCCTCGAACCCAGATGGCTTTaCGTAGAATCGAGA CCGAGGAGAGGGTTAGGGATAGGCTTACCtccgccggAGAGAACGATATCCTTC AGACGCTTCACGCTAGGAG-3'

Table S4: Primer sequences to genotype the PCDH19-V5 and KO mouse lines. For the PCDH19 KO, usage of the three primers leads to a band at 445 bp for the WT allele and 359 bp

for the mutant allele. A >1kb fragment generated by the mutant primer pair on the WT allele is usually outcompeted and not detected.

PCDH19			Sequence	%GC	bp	Tm
V5	Fwd		5'-GTGGAGCCGACAACGAGAAA-3'			
	Rev		5'-TTGCTCCAACTGAACACCCC-3'			
KO	Fwd	mut	5'-CAGAAGATTGACCGAGACCTGC-3'			
		wt	5'-CCCACACTTCTCCAAGCCTTAC-3'	55	22	57°C
	Rev		5'-GCCCTCATCATAATCGTCTGCC-3'			

Table S5: Components of probe hybridisation buffer

Component	Final concentration	Supplier
Formamide	30%	Sigma-Aldrich
SSC	5X	Invitrogen
Citric acid pH 6	9 mM	Made in house
Tween 20	0.1 %	Bio-Rad
Heparin	50 µg/mL	Sigma, cat H3393
Denhardts solution	1X	Invitrogen
Dextran sulphate	10 %	Sigma, cat D8906

Table S6: Components of probe wash buffer

Component	Final concentration	Supplier
Formamide	30%	Sigma-Aldrich
SSC	5X	Invitrogen
Citric acid pH 6	9 mM	Made in house
Tween 20	0.1 %	Bio-Rad
Heparin	50 µg/mL	Sigma, cat H3393

Table S7: Components for amplification buffer

Component	Final concentration	Supplier
SSC	5X	Invitrogen
Tween 20	0.1 %	Bio-Rad
Dextran sulphate	10 %	Sigma, cat D8906

Table S8: Components for 5X SSCT

Component	Final concentration	Supplier
SSC	5X	Invitrogen
Tween 20	0.1 %	Bio-Rad

Table S9: Probe pool for PCDH19 HCR

Pool name	Sequence
Pcdh19_B1_20PP	GAGGAGGGCAGCAAACGGAAAGTTTCGAGGAATGTAGACCTCAGC
Pcdh19_B1_20PP	CTACAGTCACCAAATAGCCTATGCCTAGAAGAGTCTTCCTTTACG
Pcdh19_B1_20PP	GAGGAGGGCAGCAAACGGAAGACCGGTGTATTGTCATTGACGTCG
Pcdh19_B1_20PP	GCCATTGATCAGAGGTGGGGCAGTGTAGAAGAGTCTTCCTTTACG
Pcdh19_B1_20PP	GAGGAGGGCAGCAAACGGAATGCAGGGAGGGCAAGCCGCCATCCT
Pcdh19_B1_20PP	ATAATGACCCTCACAGTGGCATTGCTAGAAGAGTCTTCCTTTACG
Pcdh19_B1_20PP	GAGGAGGGCAGCAAACGGAATCTGCTCATGGTTAAAGGAACGGAG
Pcdh19_B1_20PP	CCAGGACCTTGAATTCAAACGCCTTTAGAAGAGTCTTCCTTTACG
Pcdh19_B1_20PP	GAGGAGGGCAGCAAACGGAAGAGACACTGCCATTAAGACCCATGT
Pcdh19_B1_20PP	CGCACCTGTGATGGCACAATTTGATTAGAAGAGTCTTCCTTTACG
Pcdh19_B1_20PP	GAGGAGGGCAGCAAACGGAAAGGCACCAGGAGTGTTGTTCTCCTG
Pcdh19_B1_20PP	GGTCGCGGGCAGACACTGAGAGCAGTAGAAGAGTCTTCCTTTACG
Pcdh19_B1_20PP	GAGGAGGGCAGCAAACGGAAGTGTGGGTGGTTGTCATTCTCATCT
Pcdh19_B1_20PP	AATGACTTGGTAGTAAGGCTTGGAGTAGAAGAGTCTTCCTTTACG
Pcdh19_B1_20PP	GAGGAGGGCAGCAAACGGAATGCAGCATAGGCACACCGCTGTCTC
Pcdh19_B1_20PP	ATACGCACAGTGAACGACTTGGCACTAGAAGAGTCTTCCTTTACG
Pcdh19_B1_20PP	GAGGAGGGCAGCAAACGGAAGCTGCTCTCGGTCCAGCCTCCCATC
Pcdh19_B1_20PP	CCTGAATAGTGAGATTGTACTGGTCTAGAAGAGTCTTCCTTTACG
Pcdh19_B1_20PP	GAGGAGGGCAGCAAACGGAACTGCAGTCGAAAGGGGACATTGCCC
Pcdh19_B1_20PP	AAGAATAGTGGAGAAGCTCTCATACTAGAAGAGTCTTCCTTTACG
Pcdh19_B1_20PP	GAGGAGGGCAGCAAACGGAACCGGAGTCGCGATCAGACACCCGAA
Pcdh19_B1_20PP	AAGCGGCACTGCACACGTCCATTGATAGAAGAGTCTTCCTTTACG
Pcdh19_B1_20PP	GAGGAGGGCAGCAAACGGAACTCGCTATTGACCGACAGGAGGTTG
Pcdh19_B1_20PP	GGGGGCGCTCTCGCTCACCTCCACATAGAAGAGTCTTCCTTTACG
Pcdh19_B1_20PP	GAGGAGGGCAGCAAACGGAACTTGCACATCCAGTTCGTACACATG
Pcdh19_B1_20PP	GGATAGAGTTGGGCCCCAGGTCTTTTAGAAGAGTCTTCCTTTACG
Pcdh19_B1_20PP	GAGGAGGGCAGCAAACGGAAGGTGACCAGGCCGCTGTGTGGGTCA
Pcdh19_B1_20PP	CTCTTCATAGTCTAGCGCACCGGTGTAGAAGAGTCTTCCTTTACG
Pcdh19_B1_20PP	GAGGAGGGCAGCAAACGGAAACATAACCATAGAAGGAATAGACCA
Pcdh19_B1_20PP	TGGAAGAGTTCACGCGTACGGTCATTAGAAGAGTCTTCCTTTACG
Pcdh19_B1_20PP	GAGGAGGGCAGCAAACGGAATGAGTAGGTGGACTCGCCAAACACT
Pcdh19_B1_20PP	GGGAGGTGAATTTTCAGGCACGCTCTAGAAGAGTCTTCCTTTACG
Pcdh19_B1_20PP	GAGGAGGGCAGCAAACGGAAATGCTGAGGCCAACTGTGCCCATGT
Pcdh19_B1_20PP	TTGTTGTCATTCGAATCGGTCACCTTAGAAGAGTCTTCCTTTACG
Pcdh19_B1_20PP	GAGGAGGGCAGCAAACGGAATGCGAAAGCTGTAATGTGACTGTGT
Pcdh19_B1_20PP	GTGGGTCGCCTCCGTCGAGAGCCGTTAGAAGAGTCTTCCTTTACG
Pcdh19_B1_20PP	GAGGAGGGCAGCAAACGGAATTCAGCAAAGCGCGAACCGTCACCC
Pcdh19_B1_20PP	ACGGTCCAGGCTCTTCTCCACCACCTAGAAGAGTCTTCCTTTACG
Pcdh19_B1_20PP	GAGGAGGGCAGCAAACGGAATTGGGCGTGAGCTCGTAGGTCTGCA
Pcdh19_B1_20PP	GTCTTTATTTCCAGGCCAAACAGCTTAGAAGAGTCTTCCTTTACG

1.2 Supplementary Figures

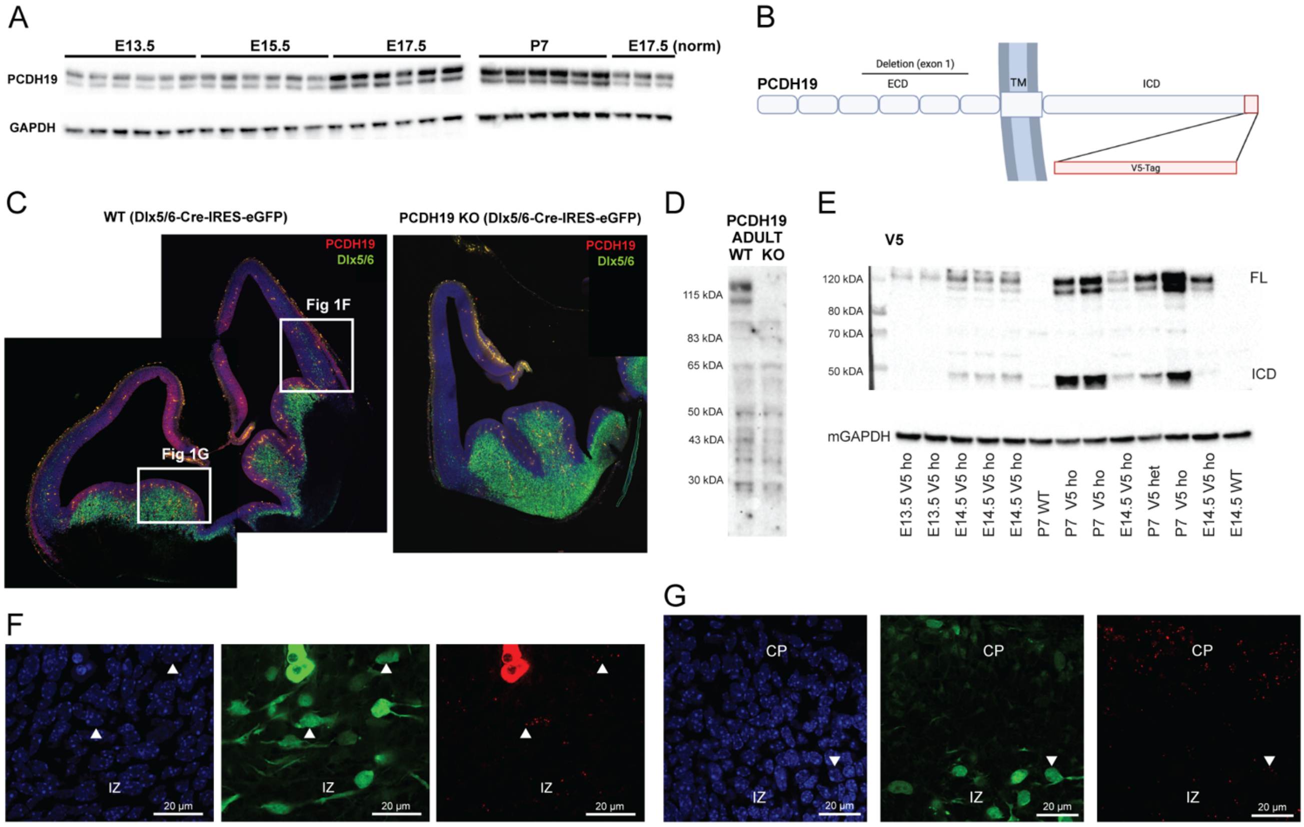

Supplementary Figure 1. PCDH19 expression, generation and validation of PCDH19-V5 and PCDH19 KO mouse lines. **(A)** Western blot of PCDH19 (Bethyl anti-PCDH19) in telencephalon lysates at different embryonic stages. Three E17.5 samples were taken along with a second WB for normalisation (E17.5 norm). PCDH19 levels increased during development. **(B)** Schematic of PCDH19 protein with depiction of deletion and the C-terminal V5 tag, used to create 2 different mouse lines. **(C)** Mosaic pictures of E13.5 coronal telencephalon sections with PCDH19 HCR (red) in Dlx5/6-Cre-eGFP (green) animals. White squares depict where pictures in Figure 1F and G were taken. Note the high to low gradient in PCDH19 expression in the hippocampus to neocortex. The PCDH19 KO section did not show PCDH19 signal. **(D)** Western blot showing absence of PCDH19 protein from an adult PCDH19 KO mouse telencephalon. **(E)** Western Blot done on the V5 tag on PCDH19-V5 mouse brain protein lysates. Multiple bands were obtained for the full-length protein, which could represent isoforms or post translational modifications of the protein. More protein can be identified at P7. A stronger band at 50 kDa could represent the cleaved PCDH19-V5 ICD domain. **(F)** Separate channel pictures of Figure 1H. **(G)** Separate channel pictures of Figure 1I.

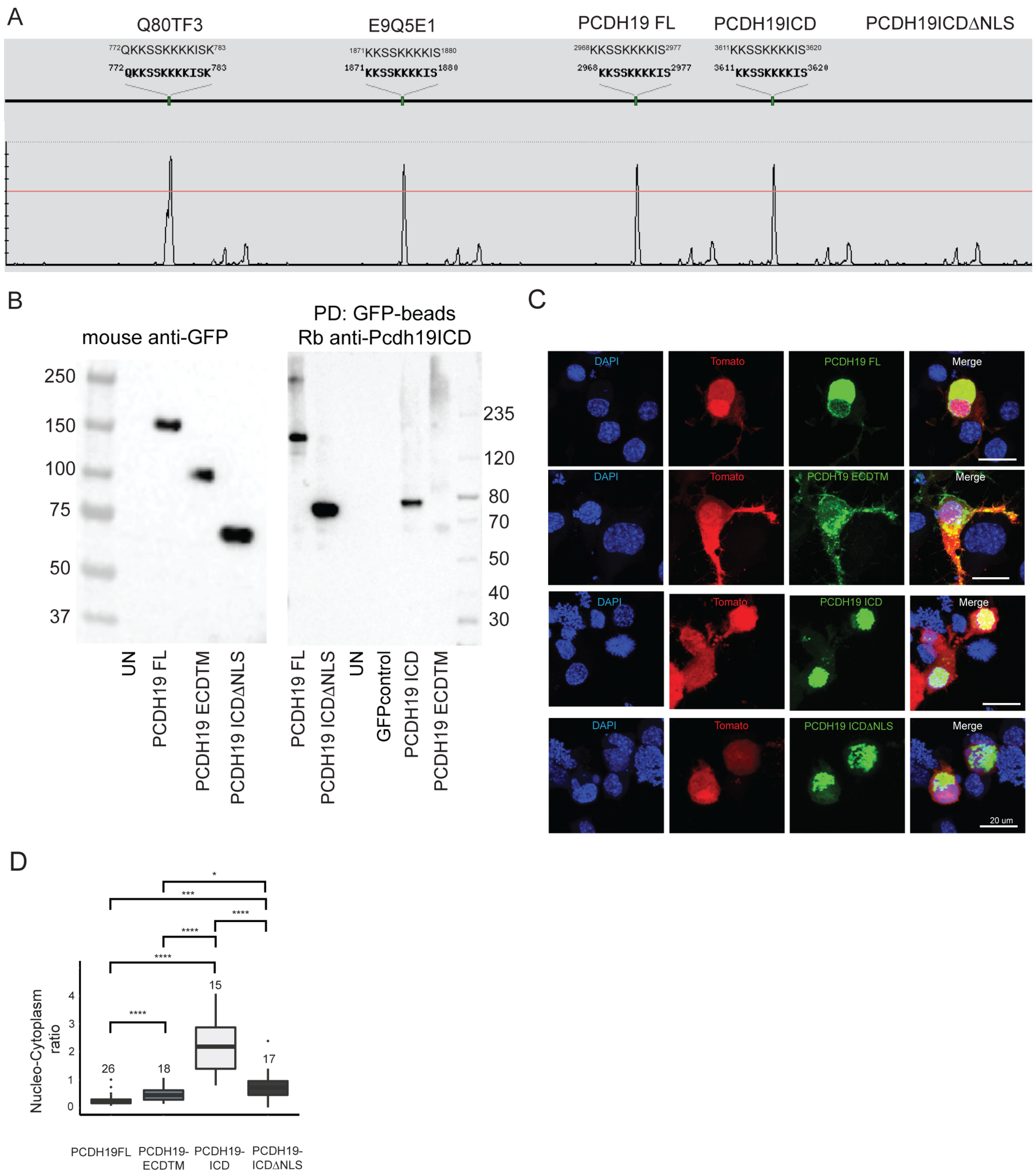

Supplementary Figure 2. Subcellular localization of PCDH19 overexpression constructs and *in vitro* production of the C-terminus GFP-tagged PCDH19 protein subdomains including PCDH19ICDΔNLS that lacks the predicted NLS. **(A)** NLStradamus prediction shows NLS in both mouse PCDH19 isoforms Q80TF3 and E9Q5E1 as well as inside our overexpression constructs, exempting PCDH19ICDΔNLS which was designed without the depicted and predicted nuclear localization signal. **(B)** Western Blot using an anti-GFP antibody that detected all the GFP-tagged subdomains at the correct molecular weight: PCDH19FL at 148.4 kDA, PCDH19ECDTM at 104.2 kDA and PCDH19ICDΔNLS at 67.7 kDA. No band was detected in the untransfected control (UN) (left blot). Western Blot result after performing a pull down on the GFP tag and detection using an antibody against the ICD of PCDH19. Bands were present for PCDH19FL, PCDH19ICDΔNLS and PCDH19ICD at 71.7 kDA. No bands were detected for the GFP control, a plasmid containing GFP but no PCDH19, for the untransfected control as well as PCDH19ECDTM that lacks the ICD. **(C)** Subcellular localization of PCDH19 in Neuro2A cells. PCDH19 subdomain

expression in Neuro2A cells 48 hours after transfection. Separate fluorescence channels as well as the overlay are shown. **(D)** Nucleo-cytoplasmic ratio quantification, ratio > 1 corresponds to preferentially nuclear localization. Ratio < 1 corresponds to enrichment in the cytoplasm, n is indicated on top of the box plots. Multiple mean comparisons using the Wilcoxon test, indicated significant differences ****p < 0.0001, ***p = 0.00023, *p = 0.01459.

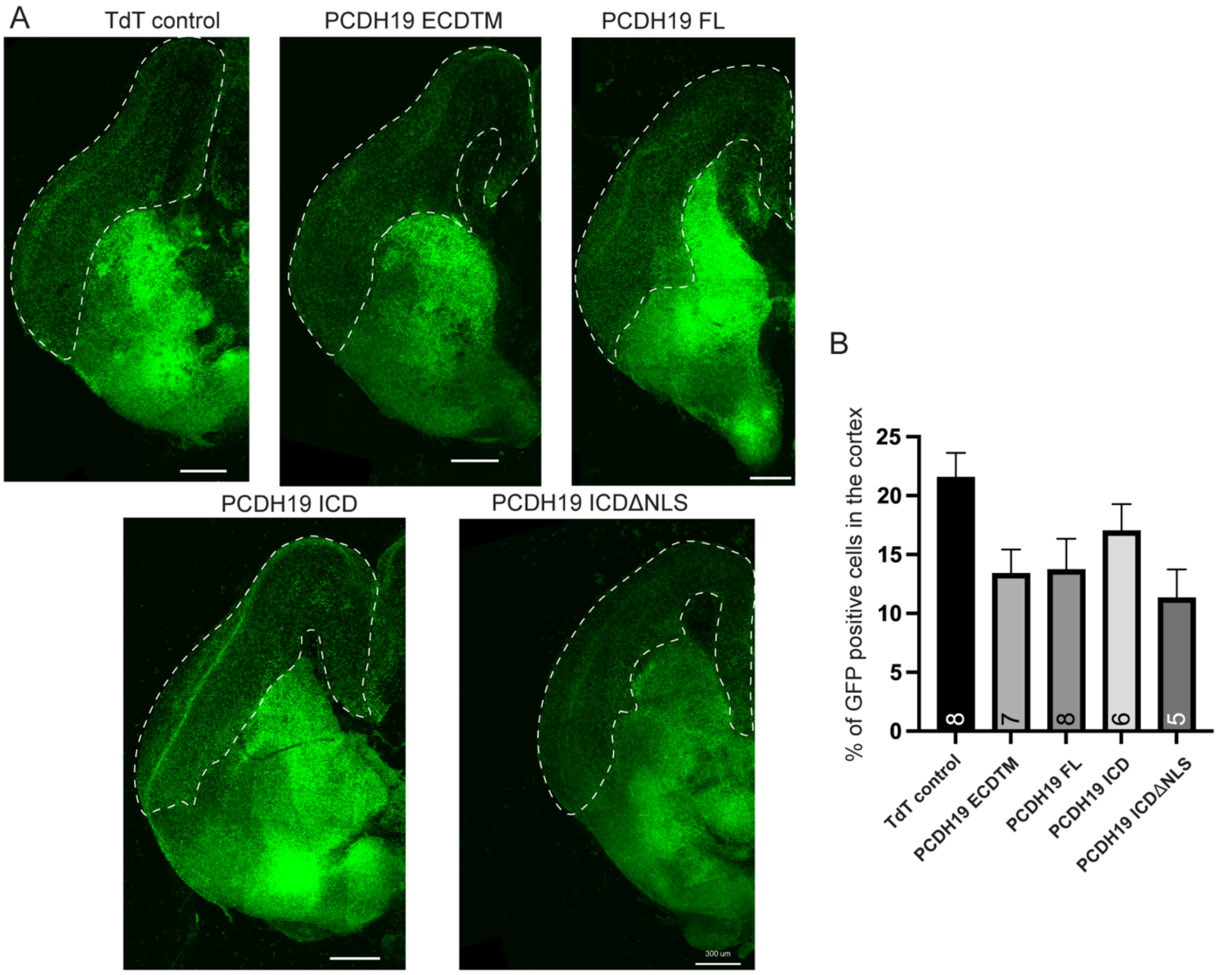

Supplementary Figure 3. PCDH19 overdosing does not have a non-cell autonomous effect on interneuron migration in the electroporated brain slices. **(A)** Z-stack example images of brain slices taken 72h post-electroporation of TdT control and PCDH19 subdomains as well as the full-length protein (PCDH19FL), the dotted line represents the cortical field. **(B)** IN migration to the cortical field was assessed by measuring the intensity of the GFP+ neurons in the cortical field divided by the intensity of the GFP+ neurons of the whole brain slice. No significant difference was measured between the means, Kruskal Wallis test.

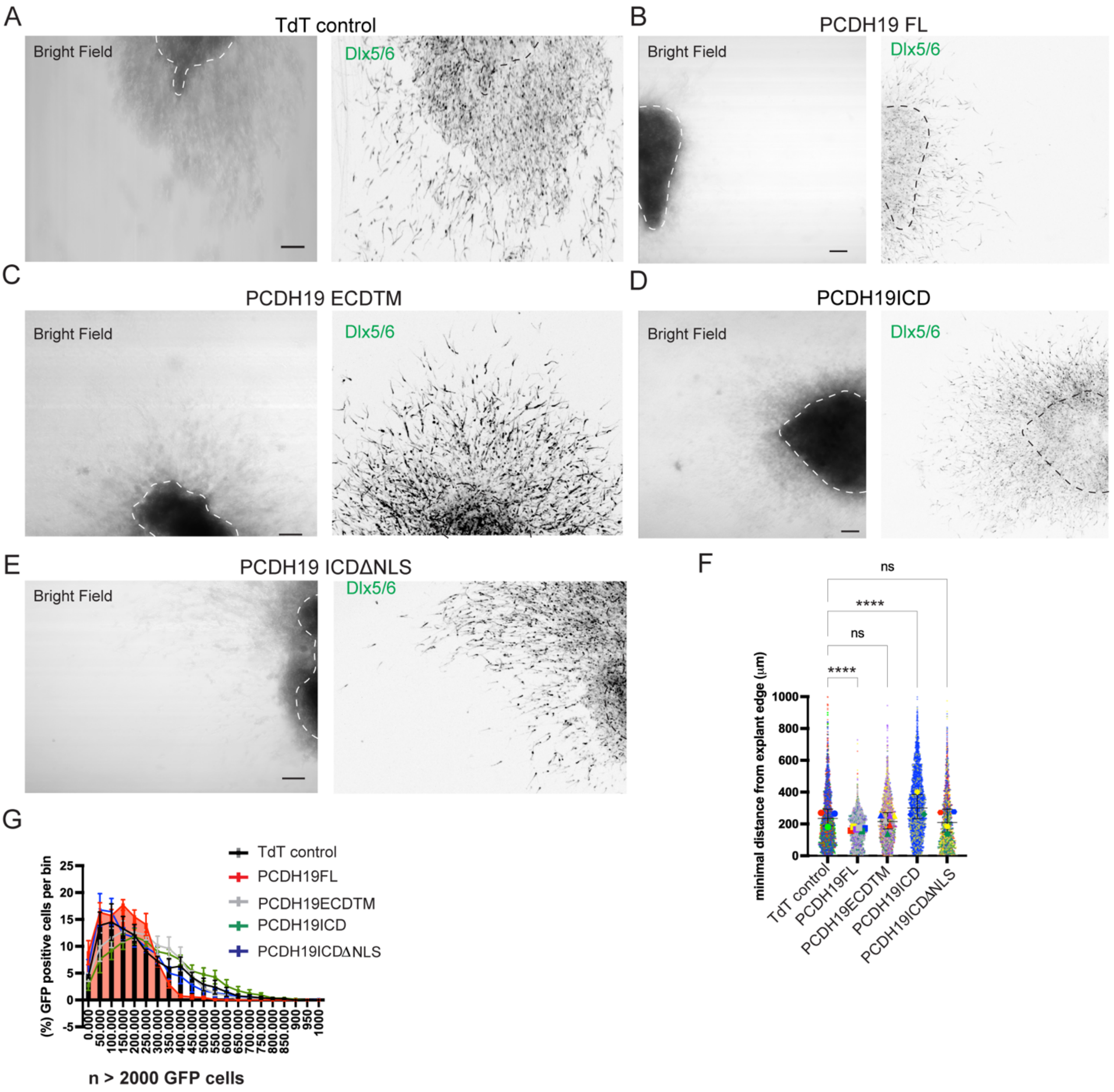

Supplementary Figure 4. PCDH19 subdomain overexpression has a non-cell autonomous effect on MGE cell migration. (A-E) Example images of electroporated and cultured explant 48 hours post electroporation of the TdTomato control plasmid (A) and PCDH19 constructs on the Dlx5/6 explant neurons (Dlx5/6), the dotted line represents the explant edge which can be visualized in the bright field image. (F) Dot plot depicting GFP^{+} IN-related minimal distance from the explant edge in TdT control and diverse PCDH19 constructs. Each dot represents one electroporated neuron in the respective condition, colors of the dots relate to different explants. Significantly shorter distance from the explant edge could be measured between PCDH19 FL and TdT control (****$p < 0.0001$) as well as a significant larger distance between PCDH19 ICD and TdT control (****$p < 0.0001$), (Kruskal-Wallis non parametric test and Dunn`s post hoc test, ****$p < 0.0001$). (**G)** Quantification of GFP neurons per bin normalized against the total amount of GFP neurons per bin showed non-significant difference per bin (Mixed model ANOVA test). Scale bar 100 μm.

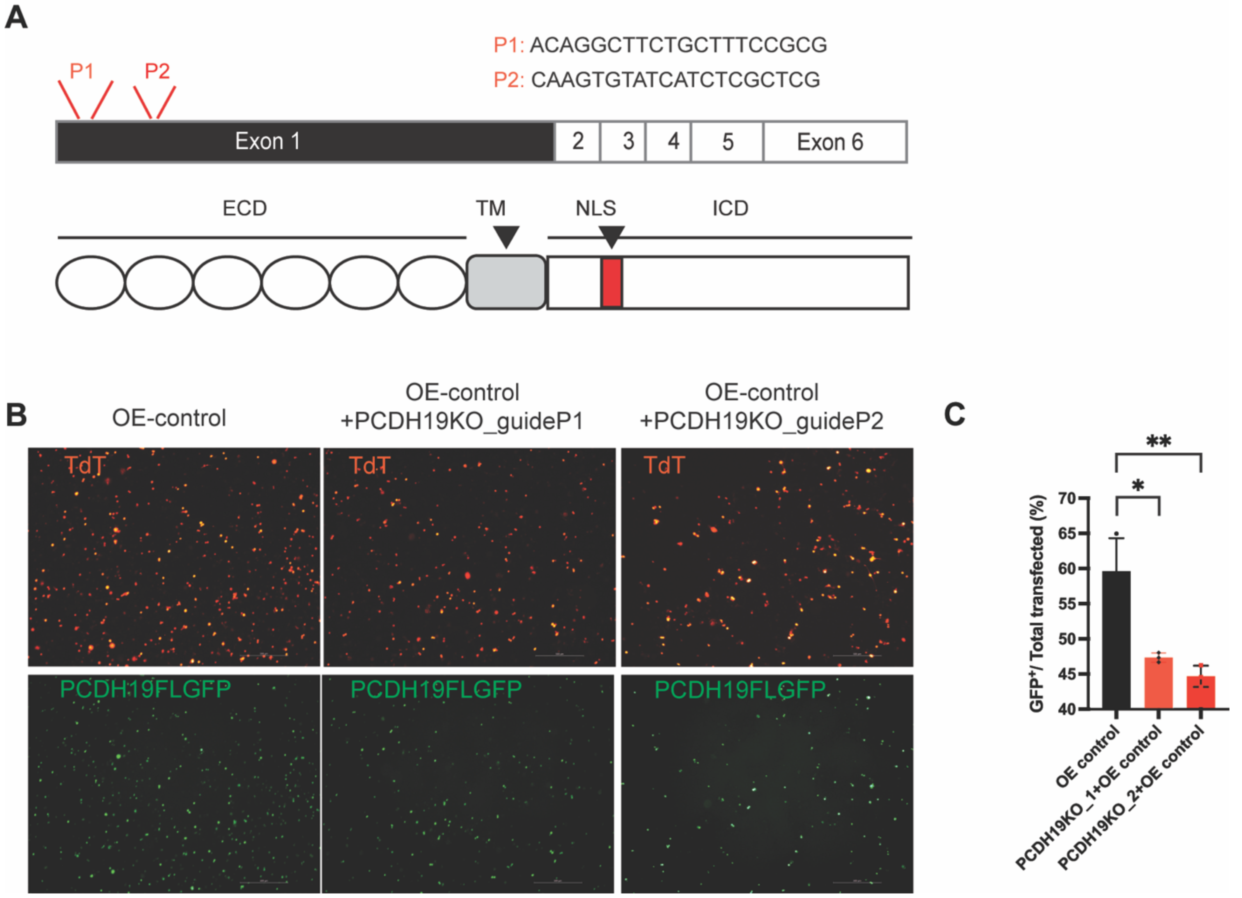

Supplementary Figure 5. Validation of PCDH19KO guides. (A) *Pcdh19* exonic structure shows location of the designed sgRNA guides, P1 and P2. P1 targets around bp 167 in exon 1. P2 targets around bp 290 in exon 1. Guide sequences are shown. (B). Neuro 2A cells transfected with the bi-cistronic overexpression plasmid of PCDH19 (PCDH19FLGFP-TdT) after 48 hours of culture. scale bars are 500µm (C). Flow cytometric analysis of cells in (B) shows significant reduction of GFP^+ cells upon CRISPR P1 and P2 KO over the total number of targeted cells compared to the control (PCDH19FLGFP-TdT plasmid only). Unpaired T-test with p** = 0.0063 and Welch T-test with p*=0.0430.

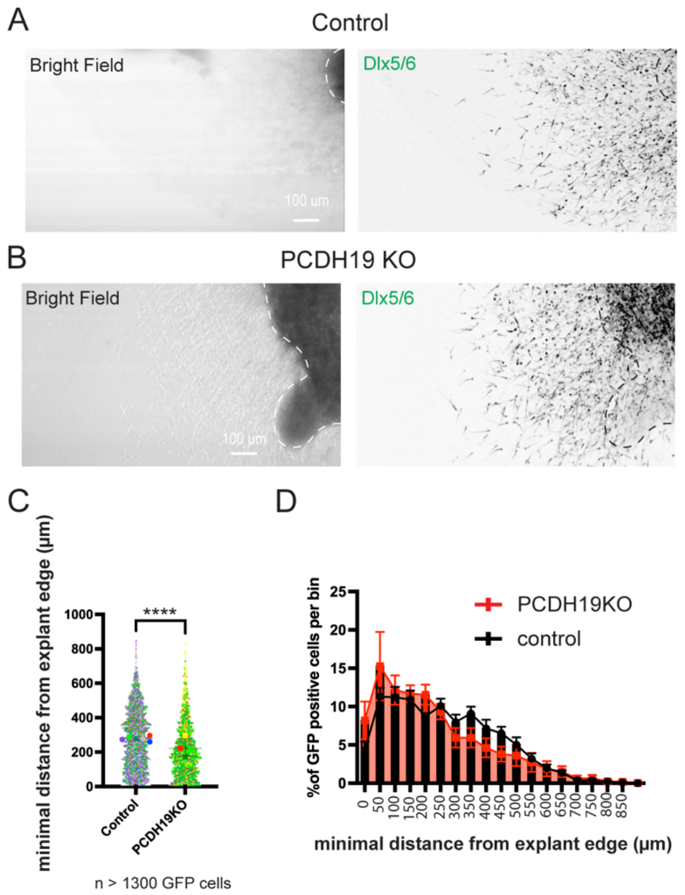

Supplementary Figure 6. PCDH19 KO has a non-cell-autonomous effect on MGE cell migration. (A-B) Representative images of electroporated and cultured explants 48 hours post electroporation of control Cas9 (A) and PCDH19KO RNP (B) of the Dlx5/6 ireseGFP neurons (Dlx5/6). (C) Dot plot depicting Dlx5/6eGFP+ IN related minimal distance from explant edge. Each dot represents one neuron in the respective control and PCDH19KO condition, replicates are depicted by color. Significant shorter distance from the explant edge was measured in the PCDH19KO compared to control (Mann Whitney U test, ****$p<0.0001$). (D) Quantification of GFP neurons per bin normalized against the total amount of GFP neurons per bin showed non-significant difference per bin (Mann Whitney U test, followed by multiple false discovery rate correction).

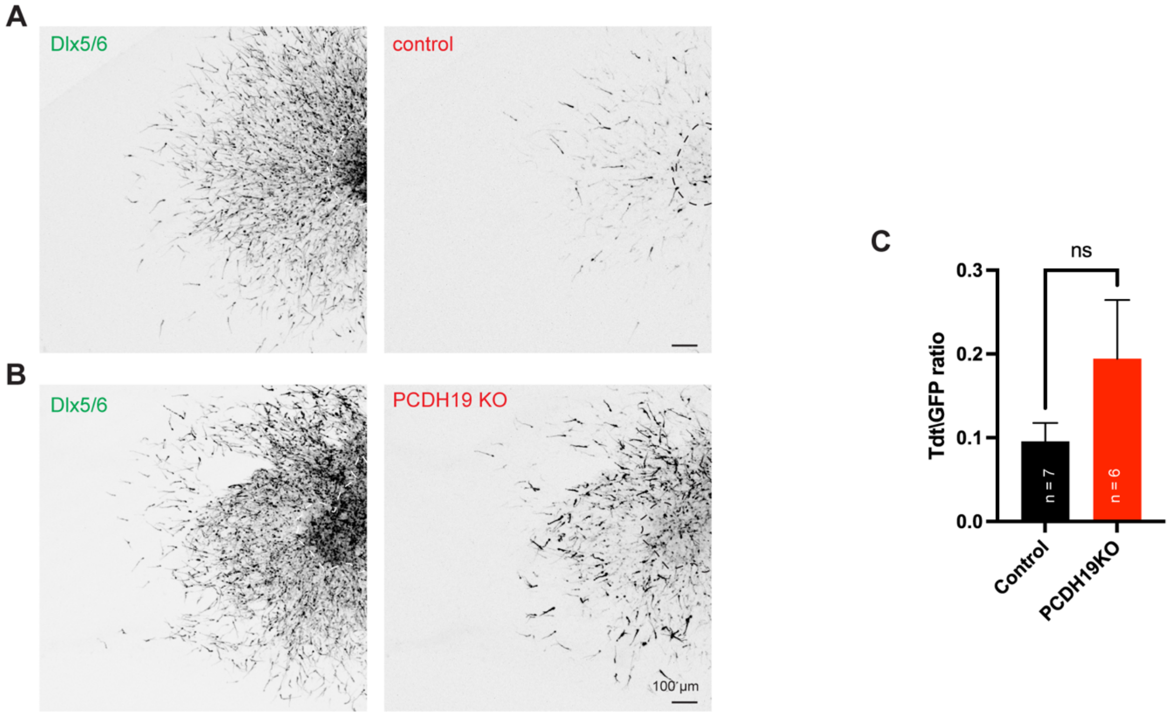

Supplementary Figure 7. PCDH19 loss of function seems to not induce apoptosis in MGE electroporated INs. (A-B) Explant examples of the control **(A)** and PCDH19KO **(B)** shown in the GFP fluorescent channel (Dlx5/6) and in the red fluorescent channel (TdT) respectively. Areas and shapes are different in the two distinct experimental conditions. The dotted line indicates the explant boundary. **(C)** Quantification of the TdTomato signal (proxy for the amount of targeted cells) relative to the GFP signal (Dlx5/6 INs) normalized to the size of the explant. A lower ratio was found in the control compared to the PCDH19 KO, but this observation was not significant (Mann Whitney U Test).
